# Traits, phylogeny and host cell receptors predict *Ebolavirus* host status among African mammals

**DOI:** 10.1371/journal.pntd.0010993

**Published:** 2022-12-21

**Authors:** Mekala Sundaram, John Paul Schmidt, Barbara A. Han, John M. Drake, Patrick R. Stephens

**Affiliations:** 1 Department of Integrative Biology, Oklahoma State University, Stillwater, Oklahoma, United States of America; 2 Odum School of Ecology, University of Georgia, Athens, Georgia, United States of America; 3 Cary Institute of Ecosystems Studies, Millbrook, New York, United States of America; 4 Center for the Ecology of Infectious Diseases, University of Georgia, Athens, Georgia, United States of America; University of Texas Medical Branch / Galveston National Laboratory, UNITED STATES

## Abstract

We explore how animal host traits, phylogenetic identity and cell receptor sequences relate to infection status and mortality from ebolaviruses. We gathered exhaustive databases of mortality from *Ebolavirus* after exposure and infection status based on PCR and antibody tests. We performed ridge regressions predicting mortality and infection as a function of traits, phylogenetic eigenvectors and separately host receptor sequences. We found that mortality from *Ebolavirus* had a strong association to life history characteristics and phylogeny. In contrast, infection status related not just to life history and phylogeny, but also to fruit consumption which suggests that geographic overlap of frugivorous mammals can lead to spread of virus in the wild. Niemann Pick C1 (NPC1) receptor sequences predicted infection statuses of bats included in our study with very high accuracy, suggesting that characterizing NPC1 in additional species is a promising avenue for future work. We combine the predictions from our mortality and infection status models to differentiate between species that are infected and also die from *Ebolavirus* versus species that are infected but tolerate the virus (possible reservoirs of *Ebolavirus*). We therefore present the first comprehensive estimates of *Ebolavirus* reservoir statuses for all known terrestrial mammals in Africa.

## Author summary

Identifying the animal hosts of *Ebolavirus* is crucial to preventing future outbreaks. We gathered exhaustive databases of which species die and which species show evidence of past infection from ebolaviruses in published literature. Our approach allowed us to differentiate which species show high mortality from *Ebolavirus* and which species tolerate the infection after exposure. We found that fruit bats are likely reservoirs as they are exposed to the infection but tolerate the virus, whereas primates do not serve as ideal reservoirs because they succumb to the infection once exposed. We also compared different predictors of infection and conclude that receptors of *Ebolavirus* best predict infection in bats whereas ecological traits predict infection in primates.

## Introduction

Ebolaviruses are zoonotic pathogens causing deadly hemorrhagic fever in human and animal populations [1]. Spillover of ebolaviruses into humans have occurred at least since 1976 with known and suspected index cases coming into contact with a wide range of possible animal hosts through hunting, transportation and eating of wild caught mammals [1–3]. Source species implicated in individual outbreaks include gorillas (*Gorilla gorilla*), chimpanzees (*Pan troglodytes*), baboons (*Papio anubis*) and several bats [4–6]. In the last few decades, much research has focused on the wild source of ebolaviruses [7–11].

Though the source of initial infection in several previous outbreaks [1,2,12], gorillas [13] and chimpanzees [14] show extremely high mortality when infected, and are, therefore, not considered to be a major source of transmission to other species [9,15]. While efforts have been made to identify a definitive mammal or arthropod reservoir [7,8,10,16], no reservoir species with high seroprevalence has yet been identified [11]. Rather than a single reservoir species, a network of maintenance hosts may be supporting circulation of ebolaviruses in the wild [11,17,18]. However, no prior study has attempted to estimate the host range of ebolaviruses across all known African mammals.

In previous work, Schmidt et al. [19] used machine learning to explore traits associated with variation in *Ebolavirus* infection status for 119 species sampled in the wild. This study found a high probability of infection for Pteropodid bats, primates and artiodactyls. Similarly, Han et al. [20] estimated the potential global host range of the Filoviridae in bats. While neither study directly incorporated phylogenetic information into models, Schmidt et al. [19] found statistically significant phylogenetic signal in *Ebolavirus* infection status. Moreover, phylogeny has been shown to be a consistent predictor of pathogen sharing among host species in other systems [21–23]. Olivero et al. [24] also concluded that Pteropodid bats, Molossid bats, primates and ungulates were phylogenetically close to known hosts and geographically linked to *Ebolavirus* outbreaks. No study thus far has quantified species-level differences in response to *Ebolavirus* infection, such as differences in susceptibility and mortality. Some species that are likely to test positive for infection tend to show high mortality when infected, while others are able to tolerate infection [19]. Presumably, the latter tolerant group includes the most important reservoirs in the wild such as Pteropodids [11,17,25] as these species will likely sustain the virus in the wild for long periods of time [11].

Intriguingly, a recent laboratory study also suggested that Niemann Pick C1 (hereafter ‘NPC1’) protein sequences may affect variation in infectivity of ebolaviruses at the cellular level [26–28], suggesting a molecular basis for host range. NPC1 is a transmembrane protein, which when functionally impaired in humans leads to lipid accumulation in cellular lysosomes and causes fatal Niemann Pick disease [29]. NPC1 has also been identified as a filovirus receptor fusing to the glycoproteins of filovirus envelopes and facilitating cell infection [26,30]. In a laboratory study of cell lines of two species of bats (specifically ZFBK13-76E and FBKT1 cell lines) and humans (HEK293T cell line), Takadate et al. [26] showed that specific amino acid residues in the loop-1 and loop-2 regions of NPC1 confer resistance to African filoviruses (*Marburgvirus* and *Ebolavirus*) by reducing cellular binding affinity of virus glycoproteins and inhibiting infection. Because of the genetic disorder that it can cause, there is much research interest in NPC1 (e.g., [31–35]), and protein sequence data for more than 100 species of mammal are available in GenBank for the binding region that Takadate et al. [26] showed to be important [36]. Whether NPC1 can predict the likelihood of infection in the wild for species sampled for ebolaviruses has not been tested.

Here, we compile data on African *Ebolavirus* infection (from antibody and PCR tests) and mortality in mammals. We statistically model infection status and post-infection mortality using mammal species traits and phylogenetic relationships. We also model infection status as a function of NPC1 amino acid sequence data. Finally, we use our best models to predict the likely host range of ebolaviruses across African mammals, differentiating species that fit the profile of secondary amplifying hosts (i.e., that succumb quickly when infected) from better primary reservoir candidates (i.e., species that do not succumb to infection), providing a quantitative assessment of the host species most likely to be involved in maintaining circulation of ebolaviruses in Africa.

## Methods

Here we outline research materials and methods. See “Supplemental Materials and Methods” (S1 Text) for additional details and rationale. We gathered exhaustive databases of species mortality after exposure to wild type strains of African ebolaviruses and species infection statuses determined from antibody and PCR tests in field survey studies. We created a binary variable of 1 for high mortality after exposure and 0 for little or no mortality. We created a second binary variable of 1 for positive infection status detected from PCR and antibody tests and 0 for species with only negative test results.

We chose life history traits describing ‘slow’ pace-of-life (or slow development and long gestation) vs ‘fast’ pace-of-life (or quick development and reproduction), which have been shown to be important in past studies of infection [19,20], from a near-complete imputed database of mammal traits [37]. We further chose brain mass as a trait representing life history tradeoffs in mammals [38]. Our final list included adult mass (g), brain mass (g), maximum longevity (d), age at first reproduction, gestation length (d), litter size, litters per year and traits reflecting variation in diet including percent diet comprised of scavenged meat, grain, fruit, and plant material. We also included distance of geographic range to a spillover site (m; computed as distance of IUCN range to nearest Schmidt et al. [39] spillover site) and a binary variable of 1 for volant and 0 for non-volant to distinguish bats from other species. For infection status models, we also used summed numbers of individuals sampled across all studies as a measure of sampling effort. To incorporate phylogenetic information, we used the maximum clade credibility tree from a recently published phylogenetic study of all mammals [40], and repeated analyses with a random sample of 100 alternative trees from the Bayesian posterior distribution of possible trees. For each tree we estimated phylogenetic eigenvectors using R package ‘PVR’ [41]. We included the first 48 eigenvectors, which captured 75% of total variation in the phylogeny, in models. We calculated phylogenetic signal in host mortality and infection status using Fritz and Purvis’ D [42] implemented in R package ‘caper’ and tested for significance using null models assuming no phylogenetic structure and a random Brownian process.

We predicted death of mammalian hosts using a logistic ridge regression analysis modified for small sample sizes. The ridge method is a penalized regression approach that typically performs well with correlated predictors [43,44]. We used a modified procedure for selecting the ridge parameter intended for models with small sample sizes [44,45], implemented with logisticRidge function in R package ‘ridge’ [46]. Analyses where the number of predictors greatly exceeds the number of observations are commonplace in genetic studies [44], and this method has been used in previous studies with as few as eight observations [47]. We predicted death of host as a function of pace-of-life traits and the first 48 phylogenetic eigenvectors. We assessed model accuracy using leave-one-out cross validation method to determine percent observations correctly predicted by our ridge model [48].

We analyzed host infection status using a logistic ridge regression implemented in a machine learning framework. We predicted the binary variable of positive antibody or PCR tests as a function of species pace-of-life traits, sampling effort across studies, and the first 48 phylogenetic eigenvectors. Parameter tuning for our ridge model was done using R packages ‘caret’ and ‘glmnet’ with repeated cross validation method, k = 5 folds, n = 5 repeats, down sampling to balance the design and with area under curve (AUC) as performance measure. During five-fold cross validation species assigned to one of five random “folds” are excluded during model fitting. The proportion of holdout species accurately predicted by the model, which is fitted to the remainder of the data, is used as a measure of expected model accuracy with new species for which data are not currently available. To ensure that this is not biased by the holdout species chosen, the procedure is repeated using species in each of the five random folds as holdouts. After model fitting, we then supplied the estimated lambda parameter to logisticRidge in R package ‘ridge’ to compute coefficient estimates, t-statistics and accompanying *p*-values for all predictors [46]. We estimated model accuracy using AUC. We further validated results with sensitivity tests for different subsets of data (e.g., all species for which we had data vs only species sampled using PCR) and by calculating relative contribution of individual predictors (details in supporting information, S1–S3 Tables). We predicted both mortality and infection status for all African mammals with final models.

We mined GenBank for NPC1 protein sequences for 300 species. We aligned sequences with NCBI’s constraint based multiple alignment tool (COBALT) [49]. We identified loops-1 and 2 of NPC1 in AliView v1.28 [50] and converted each residue into dummy variables in R with ‘fastDummies’. We predicted infection status as a function of NPC1 sequences, and then separately NPC1 sequences, distance to spillover and sampling effort using logisticRidge in R package ‘ridge’ [46].

## Results

**Fig 1:**
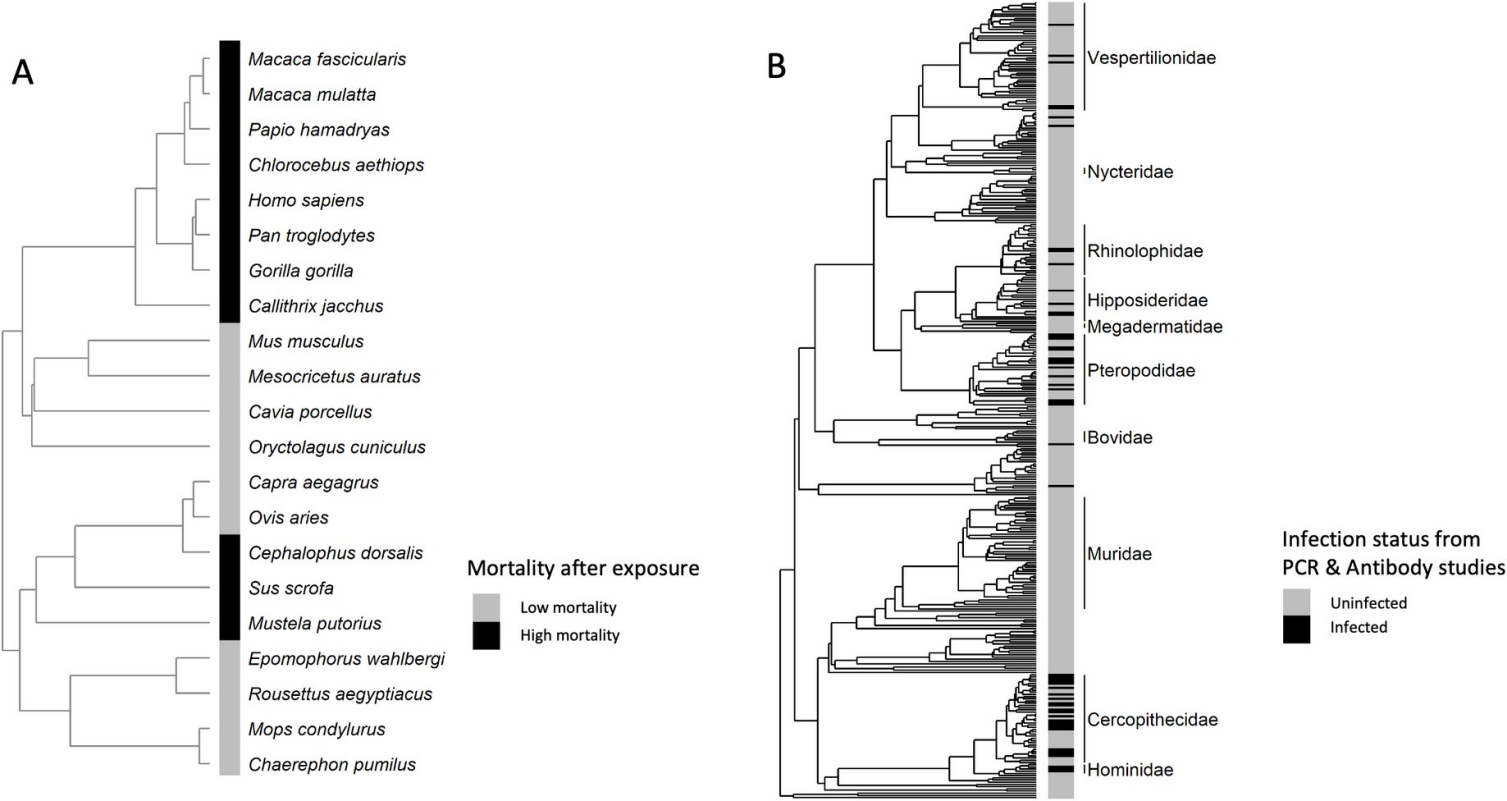
Mortality of species after exposure to *Ebolavirus* plotted on maximum clade credibility tree (A) and infection status of species determined from antibody and PCR tests plotted on maximum clade credibility tree (B).

*Ebolavirus* infection and mortality showed significant phylogenetic structure. Mortality after exposure to *Ebolavirus* (*n* = 11 species of 21 tested, Fig 1A) showed strong phylogenetic structure as measured by Fritz and Purvis’ D = -0.822. The value of D was significantly different from a random phylogenetic association (*p* = 0.001) but not significantly different from a Brownian model of evolution (*p* = 0.921) (Fig 1A). Positive infection (*n* = 56 positive species of 363 species tested) showed a weak phylogenetic signal (D = 0.482), stronger when compared to a random phylogenetic association (*p*<0.001) but less structured than a Brownian model of evolution (*p* = 0.002, Fig 1B).

We predicted mortality resulting from exposure to ebolaviruses using pace-of-life traits and phylogeny. Our ridge regression model fit to *n* = 21 species had high accuracy (~90%) using leave-one-out cross validation (Table 1). Non-volant species with long gestation lengths and fewer litters per year were more likely to die from exposure to *Ebolavirus* (Table 2). Four phylogenetic eigenvectors (c3, c6, c12 and c13) predicted the ability to tolerate *Ebolavirus* infection (Table 2; also see S2 Table). This combination of eigenvectors was also associated with a high probability of death for primates and terrestrial Artiodactyla relative to other clades (see S1 Fig).

**Table 1:** Summary of all models predicting mortality and infection status considered. For each model, table provides prediction accuracy, number of species to which model was fit, method for evaluating model accuracy and lambda parameter used in ridge regression. The two models used to predict the infection characteristics of African mammals in Fig 1 are italicized.

Model	Prediction accuracy	Number of species	Method for evaluating model accuracy	lambda
*Mortality*	*0*.*905*	*21*	*Leave-one-out CV*	*0*.*059*
*Infection status*	*0*.*802*	*363*	*AUC*	*1*.*9*
Infection status (≧10 individuals sampled)	0.765	152	AUC	2.7
PCR only	0.655	279	AUC	2.9
Infection status (only free-ranging sampled)	0.757	359	AUC	0.3
NPC1	0.645	31	Leave-one-out CV	0.094
NPC1 + distance to spillover	0.58	31	Leave-one-out CV	0.118

**Table 2:** Summary of ridge regression models. For each response variable, namely mortality, infection status, infection status for 10 or more individuals, infection status determined from PCR tests only and infection status predicted for free-ranging mammals, table summarizes the t-statistic of coefficient estimates of predictors and accompanying p-value. Coefficients for 31 eigenvectors (c1-c31) provided in table even though model was run with the first 48 phylogenetic eigenvectors; higher eigenvector coefficients for variables c32-c48 were always non-significant and therefore excluded from table. Bold faced numbers represent significance at α of 0.05. Italicized numbers represent significance at α of 0.1. NA represents coefficients that were not fit in model.

	Mortality		Infection status		Infection status (10 individuals)		PCR tested		Free-ranging mammals	
Variable	t-value	Pr (>|t|)	t-value	Pr (>|t|)	t-value	Pr (>|t|)	t-value	Pr (>|t|)	t-value	Pr (>|t|)
adult mass g	-0.379	0.705	**2.968**	**0.003**	**2.131**	**0.033**	**5.150**	**<0.001**	**1.550**	**0.121**
brain mass g	0.436	0.663	**3.321**	**0.001**	0.687	0.492	**5.979**	**<0.001**	**1.904**	**0.057**
max longevity d	1.170	0.242	**5.879**	**<0.001**	**3.213**	**0.001**	**3.731**	**<0.001**	**3.137**	**0.002**
age first reproduction d	1.185	0.236	**5.873**	**<0.001**	**3.241**	**0.001**	**4.813**	**<0.001**	**3.663**	**<0.001**
gestation length d	**2.176**	**0.030**	**6.345**	**<0.001**	**4.789**	**<0.001**	**2.820**	**0.005**	**3.798**	**<0.001**
litter size n	-0.307	0.759	**-4.104**	**<0.001**	**-3.613**	**<0.001**	-1.312	0.189	**-3.710**	**<0.001**
litters per year n	**-2.072**	**0.038**	**-4.061**	**<0.001**	**-3.298**	**0.001**	-0.270	0.787	**-1.797**	**0.072**
percent scavenge	NA	NA	-1.463	0.143	-1.096	0.273	-0.276	0.783	-1.208	0.227
percent seed	-0.414	0.679	-0.396	0.692	-1.207	0.227	-1.306	0.191	-0.316	0.752
percent fruit	0.790	0.430	**4.415**	**<0.001**	**3.275**	**0.001**	*1*.*915*	*0*.*056*	**2.636**	**0.008**
percent plant	-1.372	0.170	-0.018	0.986	0.020	0.984	0.608	0.543	-0.636	0.525
distance to spillover m	-0.026	0.979	-1.937	0.053	-1.028	0.304	-1.611	0.107	-0.922	0.356
terrestrial volant	**-2.119**	**0.034**	0.405	0.686	0.821	0.411	-0.001	0.999	**2.733**	**0.006**
c1	-0.218	0.827	**-2.014**	**0.044**	**-2.588**	**0.010**	-0.944	0.345	**-2.823**	**0.005**
c2	0.771	0.441	-1.121	0.262	-1.859	0.063	-0.504	0.614	**-2.687**	**0.007**
c3	**2.724**	**0.006**	**2.632**	**0.008**	**2.064**	**0.039**	1.243	0.214	-0.032	0.975
c4	-1.756	0.079	**-5.622**	**<0.001**	**-4.467**	**<0.001**	-1.054	0.292	**-2.367**	**0.018**
c5	-0.008	0.994	-0.628	0.530	-0.062	0.951	-0.965	0.334	-1.038	0.299
c6	**-2.474**	**0.013**	**-5.711**	**<0.001**	**-4.139**	**<0.001**	-1.515	0.130	**-2.354**	**0.019**
c7	-0.422	0.673	**-3.627**	**<0.001**	**-3.653**	**<0.001**	-1.316	0.188	**-2.640**	**0.008**
c8	0.931	0.352	0.222	0.824	0.153	0.878	0.203	0.839	0.575	0.565
c9	1.303	0.193	-0.890	0.373	-0.175	0.861	-0.324	0.746	-1.069	0.285
c10	1.426	0.154	**-3.126**	**0.002**	-1.892	0.059	-0.721	0.471	**-3.433**	**0.001**
c11	1.259	0.208	**-3.309**	**0.001**	**-2.677**	**0.007**	-0.925	0.355	**-3.497**	**<0.001**
c12	**-2.207**	**0.027**	**-4.325**	**<0.001**	**-3.610**	**<0.001**	-0.995	0.320	-1.501	0.133
c13	**2.162**	**0.031**	**4.552**	**<0.001**	**3.849**	**<0.001**	0.965	0.335	1.675	0.094
c14	-0.155	0.877	**-2.006**	**0.045**	-1.398	0.162	-0.705	0.481	-1.020	0.308
c15	1.443	0.149	**-2.455**	**0.014**	**-2.554**	**0.011**	**-2.278**	**0.023**	**-2.042**	**0.041**
c16	-0.181	0.857	-1.149	0.250	-1.623	0.104	-0.512	0.608	-0.919	0.358
c17	-0.848	0.397	1.756	0.079	1.890	0.059	0.270	0.787	1.084	0.278
c18	-0.429	0.668	-1.088	0.277	-1.518	0.129	0.395	0.693	-0.561	0.575
c19	-0.936	0.349	-1.436	0.151	-1.100	0.271	-0.998	0.318	-0.592	0.554
c20	-0.419	0.675	-1.570	0.116	*-1*.*779*	*0*.*075*	1.615	0.106	-0.874	0.382
c21	-0.862	0.388	0.218	0.828	0.391	0.696	-0.093	0.926	0.199	0.842
c22	-0.828	0.408	0.025	0.980	-0.576	0.564	**3.071**	**0.002**	0.293	0.770
c23	-0.617	0.537	1.295	0.195	0.648	0.517	**2.817**	**0.005**	1.131	0.258
c24	-0.823	0.410	0.394	0.693	0.258	0.796	0.317	0.751	0.340	0.734
c25	1.196	0.232	0.418	0.676	0.223	0.824	-0.016	0.987	0.298	0.765
c26	0.616	0.538	0.210	0.833	0.364	0.716	-0.893	0.372	-0.018	0.986
c27	0.674	0.500	-1.289	0.197	-1.117	0.264	-0.649	0.516	-0.894	0.371
c28	-0.452	0.651	**-6.373**	**<0.001**	**-4.417**	**<0.001**	-0.653	0.514	**-2.412**	**0.016**
c29	-0.370	0.711	-1.377	0.169	-1.358	0.174	-0.383	0.702	-1.016	0.310
c30	-1.000	0.317	0.299	0.765	-0.257	0.797	0.206	0.836	0.272	0.786
c31	-0.737	0.461	-1.877	0.061	**-2.011**	**0.044**	-0.624	0.533	-1.464	0.143
sample size	NA	NA	**6.110**	**<0.001**	**3.445**	**0.001**	**2.986**	**0.003**	**6.045**	**<0.001**

Infection status was related to pace of life traits, fruit consumption and phylogeny. The final model fit had high accuracy with an estimated AUC of 0.802 (Table 1). Species with large adult body mass, large brain mass, high longevity, older age at first reproduction, long gestation length, small litter sizes and fewer litters per year were more likely to test positive for *Ebolavirus* infection than other species across all subsets of data (Table 2). Furthermore, species with high percentage fruit in their diet and species sampled extensively also tested positive for ebolaviruses (Table 2). Multiple phylogenetic eigenvectors were significant predictors of infection status (Table 2) with strong support for three eigenvectors (c3, c11 and c12, S3 Table), corresponding to species in the families Cercopithecidae and Hominidae. Furthermore, fruit bats in the family Pteropodidae showed high infection probabilities (see S1 Fig).

We found NPC1 sequences predicted infection status in bats with high accuracy and that key amino acid positions thought to confer resistance to filoviruses were significant predictors of infection status. Mutations in loop-1 at positions 425–427 previously found to confer resistance to *Marburgvirus* [26] were related to infection status in 31 species for which both sequence and infection status data were available (Tables 1, S4, and S5). Specifically, absence of residue T, E and T in positions 425, 426 and 427 was related to higher probability of infection (S4 Table). Furthermore, residue A at position 425 and G at 426 was positively related to infection; these residues are believed to control susceptibility to *Marburgvirus* in laboratory analyses of bat cell lines [26] (S4 Table). Even models of NPC1 with nuisance variables of sampling effort and distance to spillover site still found significant positive infection in species with residue A at position 425 and marginal significance for other residues in positions 425–427 (S4 Table). Although our NPC1 models showed poor accuracy using leave-one-out cross validation (Table 1), these models predicted infection status of bats with 100% accuracy; whereas trait and phylogenetic models showed only 71% accuracy (Table 3) likely due to the low sensitivity of trait and phylogenetic eigenvector models for this group, meaning the ability to identify a true positive infection status in bats is low (see S6 Table). For primates, the percent accuracy of all models was low at 50–58% (Table 3); however, the sensitivity of trait and phylogenetic eigenvectors models was 1 (S6 Table). Therefore, the low accuracy was due to low specificity (i.e., poor ability to identify a true negative infection status) for primates (S6 Table).

**Table 3:** Proportion of correct predictions for species in orders Chiroptera and Primates made by three competing models, namely trait and phylogenetic eigenvector model, NPC1 sequence model, and NPC1 model with distance to spillover and sampling effort variables included.

	Proportion correct predictions	
	Chiroptera (n = 14)	Primates (n = 12)
Trait + Phylogenetic eigenvectors	0.714	0.5
NPC1	1	0.583
NPC1 + distance to spillover	1	0.583

**Fig 2:**
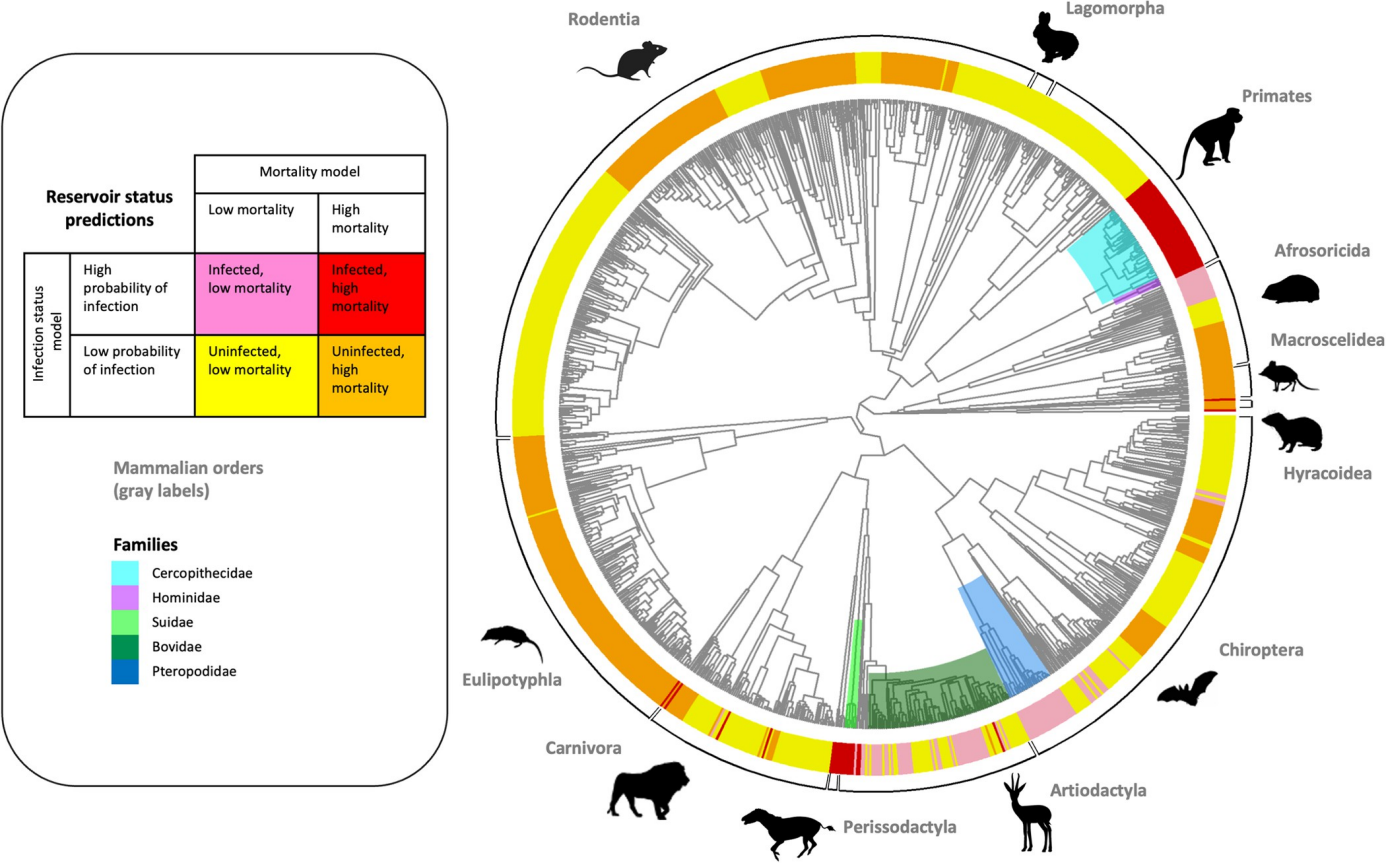
Predictions of reservoir status for all terrestrial African mammals based on ridge model predicting mortality of species after exposure to *Ebolavirus* and ridge model predicting infection status of species. Ridge models used trait and phylogenetic eigenvectors as predictors (see main text for more details of models). Silhouettes used are available under Public Domain (https://creativecommons.org/publicdomain/zero/1.0/).

Reservoir status predictions from our ridge regression models showed strong correlations to phylogenetic clades (Fig 2, see S2 Fig for visual comparison of predictions with raw data). The order Perissodactyla, families Ceropithecidae, Hominidae and Suidae showed high likelihood of death after exposure to *Ebolavirus* as well as high probability of past infection as estimated by antibody and PCR tests (Fig 2). We interpreted this category as ‘dead-end hosts’ unlikely to survive after exposure to *Ebolavirus* and therefore unlikely to sustain the pathogen for long periods of time in the wild; a criteria typically considered to be important for a species to serve as a ‘natural reservoir’ for a pathogen [11]. In contrast, fruit-eating bats in family Pteropodidae showed high probability of past infection in antibody and PCR tests and are predicted to have low mortality following exposure (Fig 2). Some members of Bovidae and Afrosoricida also fall into this category. We interpreted this group as potential ‘reservoirs’, rarely succumbing to infection and periodically serving as a source of infection for other hosts with higher mortality. Some species showed both low likelihood of being infected and low mortality even if exposed, which we interpreted as ‘low exposure and susceptibility’ (Fig 2); others showed low probability of infection but high mortality when exposed, which we interpreted as species ‘susceptible but rarely exposed’ to ebolaviruses (Fig 2). These predictions, however, are based on our trait and phylogenetic eigenvector models which has uneven prediction accuracy across clades (S6 Table).

## Discussion

Here, we explore *Ebolavirus* host range across African mammals by combining previously disparate information about host traits and phylogeny, host receptor sequences, and their ability to predict varying degrees of susceptibility to infection. This approach enabled us to determine which trait and phylogenetic (hereafter TP) variables were the best predictors of both host infection probability and mortality when infected, and to estimate the reservoir status of all terrestrial African mammals.

*Ebolavirus* mortality across species was best predicted by phylogeny and pace-of-life traits. Species vary in pace-of-life along a continuum from slow to fast; some species reproduce rapidly and have shorter life spans, while others reproduce slowly and have longer life spans [51,52]. Fast vs slow species also tend to invest differently in immune functions [53–55]. Our analyses suggest that slow species, particularly primates, with long gestation lengths and few litters per year, were more likely to succumb to infection from *Ebolavirus* (Fig 1A and Table 2). Volant species and fast paced species, such as mice (*Mus musculus*), were more likely to survive (Fig 1A and Table 2), supporting one theory that these species regulate inflammatory immune defenses to fight viral infections [53]. Several studies also suggest that bats have specific immune strategies to fight infections which could allow these species to serve as reservoirs for viruses [56]; including one study providing evidence of bat responses to *Ebolavirus* [57].

One limitation of our mortality analysis was the relatively low number of species that we could include due in large part to our reliance on laboratory studies. Among 182 observations of mortality in the wild or the laboratory that we located (see raw data provided on figshare https://doi.org/10.6084/m9.figshare.20250408.v1), a total of only 21 species were represented. Despite including relatively few observations, based on delete-one cross validation our model was able to predict the mortality of species excluded during model fitting with better than 90% accuracy (Table 1). We speculate that this high accuracy is due to a number of factors. First, we were only attempting to predict mortality at an extremely coarse level, “high” or “low.” Second, mortality once exposed to *Ebolavirus* likely depends largely on inherent characteristics of species and phylogenetic relationships, making it easier to infer mortality compared to infection status which depends on both the susceptibility of species to infection and the frequency with which they happen to encounter the virus in the wild. Finally, the ridge regression method we used [44, 45] was designed for the precise use case of an analysis where the number of predictors exceeds, or even greatly exceeds, the number of observations. Regardless, our study points to a great need for direct observations of variation in mortality after exposure to *Ebolavirus* for additional species.

Infection status was related to a suite of phylogenetic, evolutionary and ecological traits as well as to sampling effort. In contrast to mortality, frugivory and sampling effort, rather than pace-of-life traits and phylogeny alone (Table 2) were important predictors of infection status (S1 Table). This suggests that the geographic overlap of frugivorous species may support *Ebolavirus* spread among hosts in Sub-Saharan Africa. The importance of sampling effort in predicting infection from *Ebolavirus* (also noted in [19]), underscores the need for more systematic sampling across taxa, which may benefit from focused sampling during times of year when synchronous fruiting supports the spatial overlap of multiple frugivorous species that are potential hosts for ebolaviruses.

Though the data on infection status that we present represent the most comprehensive collection of *Ebolavirus* host records we are aware of, these data still have important limitations. The infection status data are likely biased as a result of better surveillance and increased sampling efforts for charismatic species or species of conservation concern. This issue could be mitigated by more systematic and focused sampling efforts in the future. Our data can also be used to identify clades that are undersampled (see S6 Table for sample sizes) and used to guide future efforts. To account for differences in sampling effort, we incorporated total number of individuals sampled as a covariate in our model and we have used repeated cross validation in a machine learning framework to test the robustness of our results. We also note that infection status determined from PCR tests have slightly different interpretations from antibody tests. Specifically, positive PCR samples suggests active circulation or infection from *Ebolavirus*; whereas positive antibody tests cannot distinguish between current or chronic infection and past exposure and infection which has been cleared [58]. Our goal was to model which types of species are likely to be susceptible to infection and therefore included both tests in most of our models. We also modelled PCR tested individuals separately and found qualitatively similar results to models including evidence of infection from any source (Table 2). The primary difference was weaker statistical support as a result of much lower numbers of species with positive PCR tests (Table 1, see S1 Text).

Infection status in bats is likely related to their immune system characteristics, whereas ecological traits of primates play a role in exposure and infection. In particular, our estimates of true positive rates, while high for the frugivorous Pteropodidae, were low for species in the insectivorous bat families Hipposideridae, Vespertilionidae, Molossidae and Minopteridae (see S6 Table). That our NPC1 models predicted the infection status of bats from multiple families with high accuracy (Tables 3 and S5) suggests that available bat trait and phylogenetic data do not adequately capture cross-species differences in immune functioning that can explain infection status. Better data on traits related to immune functioning in bats is likely to improve predictive models of infection status. Unlike bats, primates showed high true positive and low true negative rates (S6 Table). Our models predicted all Cercopithecids and Hominids are susceptible to *Ebolavirus* infection (Fig 2) even though infections have so far been documented in only a handful of species. Cercopithecid and Hominid species are also known to succumb to infection once exposed to ebolaviruses [9,15,59]. Temporal and spatial overlap with bats in the use of resources such as fruit trees may explain differential risk of exposure to ebolaviruses in the wild. Detailed behavioral data is hard to find and difficult to gather, but, nonetheless, a critical need given that seroprevalence data captures those species that can be immunologically infected with *Ebolavirus* only as a subset of species that have the ecological opportunity to be infected.

Although the estimated accuracy of our NPC1 model was low across mammals overall (Table 1), the final model successfully predicted infection status of bat species included in the model with high accuracy (Tables 3 and S5). Thus, exploring how NPC1 sequences relates to infection status in bats may improve prediction, especially given the low sensitivity of the alternative TP model. We speculate that the high accuracy of the bat predictions in the NPC1 model may be related to the fact that the loop regions we included in our models were identified by laboratory work performed on bat cell lines [26]. Further, it could also be because immunological characteristics influence infection statuses of bats to a strong degree. Our NPC1 model was less accurate for primates, but similar to the TP results, this was due to low true negative rates for primates (S5 Table) which suggests that behavioral and ecological characteristics of some primates could lower exposure to virus. Infected primates could also frequently die before they are sampled leading to negative infection statuses.

In our NPC1 model, residues that confer high affinity to binding with wild type filovirus glycoproteins strongly determined infection status. We found that residues relating to increased affinity to *Marburgvirus* [26] predicted positive *Ebolavirus* infection in bats (S4 and S5 Tables). However, the specific positions and residues identified by Takadate et al. [26] as binding to *Ebolavirus* do not match our findings. Although straw-colored fruit bats (*Eidolon helvum*) have been found to carry antibodies to *Ebolavirus* in serological studies [60–62], Takadate et al. [26] considered this species to be resistant to *Ebolavirus*. Conversely, an important reservoir for *Marburgvirus*, *Rousettus aegyptiacus* [63,64], does not carry the residues thought to confer resistance, carrying instead residues that increase affinity to the virus [26] (S5 Table). Furthermore, when inoculated with *Marburgvirus*, *R*. *aegyptiacus* tolerates infection and sheds virus [65,66] suggesting the operation of other mechanisms beyond its NPC1 receptors that allow it to fight infection [57]. This discrepancy of *R*. *aegyptiacus* not carrying the sequences needed to confer resistance to *Ebolavirus* and *Marburgvirus* was noted by Takadate et al. [26] in their study also leading to the conclusion that unique host factors such as interferons likely influence susceptibility to infection. While the positions and residues identified by Takadate et al. [26] are important predictors of binding affinity to filoviruses and possibly infection status, they do not necessarily confer resistance to filoviruses. Kurosaki et al. [67] also showed that small mutations, affecting only two amino acid residues in *Zaire ebolavirus* compared to wild type strains, can greatly increase infectivity across cell lines expressing NPC1 sequences found in both bats and primates, including humans. More work is clearly needed to identify how binding affinity to NPC1 and host immune responses relates to viral replication within an infected individual (e.g., [26,57]). Sequencing NPC1 gene regions for additional species could also be useful. For example, laboratory *Ebolavirus* inoculation studies for bat species including *Epomophorus wahlbergi* and two *Tadarida* species have been published [25], however, their NPC1 gene regions have not yet been sequenced. NPC1 sequences are currently available in GenBank for only 31 of the 363 species for which antibody and PCR test results have been published.

We modeled reservoir status across terrestrial African mammals. If we define a reservoir as a species likely to be infected but not to die, our models predict Pteropodid fruit bats, also identified as strong reservoir candidates in other studies [68], as likely reservoirs (Fig 2). In experimental inoculations of fruit and insectivorous bats with ebolaviruses, no evidence exists of either death or illness in bats carrying the virus [25], strongly supporting the idea that bats can naturally tolerate the virus while also serving as a source of infection [11,17]. Our analysis also highlights species from the order Afrosoricida and family Bovidae as potential reservoirs. To our knowledge, none of these species have been sampled for *Ebolavirus* in the wild, though some of them do overlap with known spillover locations. Interestingly, shrew species from the clade Soricidae show evidence of inserted filoviral elements in their genome, which suggests evolutionary history with filoviruses and potential infection of an ancestor [69] and positive infection status has been noted in species such as *Sylvisorex ollula* [70]. Our model is unable to predict positive infection status for this species (S6 Table). Therefore, closer examination of the clade Soricidae could also help clarify host status predictions estimated in this paper. Our analyses identified Cercopithecidae, Hominidae, Suidae and the order Perissodactyla as “dead-end”, or secondary amplifying hosts that succumb to infection rapidly. While much research has focused on identifying the elusive reservoir of *Ebolavirus* [18], known dead-end hosts including *Pan troglodytes* and *Gorilla gorilla* appear to be the source of several human outbreaks [4,15,71,72], and have also suffered drastic population size reductions after epizootic outbreaks [13,73]. Provisionally, because more testing is still needed to confirm *Ebolavirus* mortality, our study adds species from the order Perissodactyla to the list (Fig 2) of potential dead-end amplifying wild hosts. Better understanding of potential reservoir statuses across mammals (list of reservoir statuses from our models provided on figshare: https://doi.org/10.6084/m9.figshare.20250408.v1) will help further refine knowledge of the risk factors for African *Ebolavirus* spillover into human populations.

## Supporting information

S1 Text Supplementary Methods and Results. (PDF)

S1 Table Relative importances of predictor variables in ridge models. (PDF)

S2 Table Sensitivity of mortality of animal host model to uncertainty in phylogenetic relationships. (PDF)

S3 Table Sensitivity of infection status model to uncertainty in phylogenetic relationships. (PDF)

S4 Table Ridge regression predicting infection status of animal host on the basis of Niemann-Pick C1 amino acid residues, distance to spillover site and sampling effort. (PDF)

S5 Table Niemann-Pick C1 residues at key positions identified by Takadate et al. [26] for species for whom infection status is also known. (PDF)

S6 Table True positive rates (or sensitivity) and true negative rates (or specificity) provided for trait and phylogenetic eigenvector ridge model predicting infection status by mammalian clade. (PDF)

S1 Fig Plots of significant phylogenetic eigenscores for all mammalian clades. Phylogenetic eigenvector 3 or c3 plotted on maximum clade credibility tree (A), phylogenetic eigenvector 11 or c11 plotted on maximum clade credibility tree (B), and phylogenetic eigenvector 12 or c12 plotted on maximum clade credibility tree (C). These eigen scores consistently predict host mortality and host infection status; with c3 positively being related to host status, c11 and c12 being negatively related to host status.
(PDF)

S2 Fig Predictions of reservoir status for all known terrestrial African mammals and accompanying accuracy metrics. (A) Predictions of reservoir status which are based on ridge model predicting mortality of species after exposure to *Ebolavirus* and ridge model predicting infection status of species. Ridge models used trait and phylogenetic eigenvectors as predictors (see main text for more details of models). (B) Accuracy of infection status predictions by mammal clade. Silhouettes used are available under Public Domain (https://creativecommons.org/publicdomain/zero/1.0/).
(PDF)
