# Supplementary material for: Traits, phylogeny and host cell receptors predict Ebolavirus host status among African mammals
Source: PLoS Negl Trop Dis. 2022 Dec 21;16(12):e0010993. doi: 10.1371/journal.pntd.0010993 (PMC9815631; doi:10.1371/journal.pntd.0010993)
Supplement: S1 Text — (PDF) [file pntd.0010993.s001.pdf]

Supplementary S1 Text for "Traits, phylogeny and host cell receptors predict *Ebolavirus* host status of African mammals"

Mekala Sundaram, John Paul Schmidt, Barbara A. Han, John M. Drake, Patrick R. Stephens

S1 Text. Supplementary Methods and Results

Supplemental Materials and Methods

<u>*Data sources*</u>

Mortality: We compiled information from inoculation studies and field-based surveys of natural mortality to determine the ability of different mammal species to tolerate *Ebolavirus* infection. Using the search terms 'Ebola' or 'Filovirus' and 'inoculation experiment', we found 149 experimental inoculation or field-based studies of *Ebolavirus* up to the year 2021. Of these, death in individual animals from *Ebolavirus* was recorded in 103 studies. For each study, we recorded species, sample size, inoculation dose, virus strain, mortality, extent of illness and viral titer in different organs, if available. Based on multiple laboratory experiments [1,2] and field observations [3,4], *Ebolavirus* is considered to be unusual among viruses in that most mammal species either show extremely high or extremely low mortality when infected (often approaching 100% in either case). We created a binary variable of 1 for high mortality after exposure, which included >60% mortality observed in laboratory studies and estimates of mortality in the wild ≥25%; the binary variable = 0 for little or no mortality after exposure (<10% for all species). The threshold for "high mortality" was set lower for species sampled in the wild because of the difficulty in determining the cause of mortality, and the difficulty in estimating the number of individuals exposed to the virus (e.g. [4,5]). In analyses, we removed studies where experimental inoculation was performed with *Reston ebolavirus*, which is endemic to southeast Asia and exhibits much lower pathogenicity than African Ebolaviruses [6–8]. We further excluded studies in which pathogenicity was only observed with strains created using repeated serial laboratory infections of mice and guinea pigs to create mouse and guinea pig-adapted *Ebolavirus* strains. We included studies performed on carcasses of gorilla, duiker (subfamily Cephalophinae), and chimpanzees that showed strong evidence of high mortality from exposure to *Ebolavirus* (e.g. [5]). Although most inoculation experiments focused on *Zaire* and *Sudan ebolavirus*, our data captures deaths following exposure to all known wild type African Ebolaviruses including *Zaire ebolavirus*, *Sudan ebolavirus*, *Bundibugyo ebolavirus*, *Taï forest ebolavirus* and *Ivory Coast ebolavirus* (raw data provided on figshare: https://doi.org/10.6084/m9.figshare.20250408.v1).

Infection status: We searched the literature for studies documenting infection status in mammal hosts via either PCR or antibody tests. Using the search terms 'Ebola' or 'Filovirus' and 'host' or 'reservoir', we compiled 974 rows for which species-level identification of taxa was possible up to the year 2021. We included data from the PREDICT database, a large-scale effort to identify zoonotic viruses in mammals (http://data.predict.global, accessed & downloaded on 14th April 2021). Out of 974 observations, positive infection status was recorded 120 times. For each study, we compiled species identity, sample size, type of test (PCR, antibody, or viral assay), number of positive tests, location, and whether the observed animal was captive or wild. Response was scored as 1 for positive test of infection and 0 for negative test of infection (raw data provided on

figshare: https://doi.org/10.6084/m9.figshare.20250408.v1). Again, we excluded observations of *Reston ebolavirus*, since infections have only been documented outside of Africa (in the Philippines and nearby southeast Asian countries) and because this virus is known to occur in domesticated and captive species where it shows low pathogenicity [6–8].

Animal host traits: We used COMBINE [9], a mammal macroecological trait database compiled from multiple sources, including Phylacine [10], EltonTraits [11], PanTHERIA [12], TetraDENSITY [13], and MammalDIET2 [14], with good coverage across mammals. To provide nearly complete trait information for 21 traits including some related to pace-of-life such as female age at first reproduction, litter size, and maximum longevity, Soria et al. [9] imputed missing values, which we incorporated into our analyses. Pace-of-life traits capture mammal life history strategies [15], and previous studies have shown pace-of-life traits to be predictors of the mammal species in which filovirus and other zoonotic infections have been found [16–18]. We chose traits from COMBINE that were imputed with low estimated error and were nearly complete across mammal species, focusing on pace-of-life traits [15], and brain mass, which has also been shown to reflect life history trade-offs [19]. We also included dietary traits since Schmidt et al. [16] found frugivory to be an important predictor of infection status. Filtering by these criteria, we included (i) adult mass (g), (ii) brain mass (g), (iii) maximum longevity (d), (iv) age at first reproduction (d), (v) gestation length (d), (vi) litter size, (vii) litters per year and traits reflecting variation in diet including percent diet comprised of (viii) scavenged meat, (ix) grain, (x) fruit, and (xi) plant material. We also included (xii) distance of geographic range to a spillover site (m; computed as distance of the centroid of the IUCN range map to the nearest spillover site in [20]) and (xiii) a binary variable of 1 for volant mammal and 0 for non-volant mammal to distinguish bats from other species. For infection status models based on antibody and PCR tests, we (xiv) summed the number of individuals sampled across all studies as a measure of sampling effort.

Phylogeny: We used estimates of host phylogenetic relationships from a recent global study of all mammals [21] based on a fully resolved molecular tree built from published molecular data from approximately 4500 species to which remaining species were added randomly based on their taxonomic relationships to create a Bayesian posterior distribution of potential fully resolved trees. We performed preliminary analyses using the maximum clade credibility tree, the tree with the overall highest posterior probability. Results in the main paper are based on this maximum clade credibility tree. To assess the robustness of our results to uncertainty in the relationships of species incorporated based solely on taxonomy, we repeated our analyses using a random sample of 100 trees from the full posterior distribution. See section titled 'Robustness of results to phylogenetic uncertainty' for more information on methods incorporating samples of trees. To incorporate phylogenetic information into our models, we used the R package 'PVR' [22] to decompose the full mammal phylogeny into eigenvectors. The first 48 eigenvectors of the maximum clade credibility tree cumulatively explained 75% of the total variation in the phylogeny.

NPC1 genetic sequences: We mined orthologs for Niemann-Pick C1 (NPC1) protein from GenBank for all mammal species. We performed a search for NPC1 protein sequences using NCBI's Eukaryotic Genome Annotation pipeline and RefSeq platform to identify all orthologs for vertebrate species uploaded to GenBank [23]. We then used NCBI's Constraint-based

multiple alignment tool (COBALT) to align all protein sequences [24,25] and visualized sequences with AliView v1.28 [26] (complete alignment and protein accession numbers available on figshare: https://doi.org/10.6084/m9.figshare.20250408.v1). We identified the loop-1 and loop-2 amino acid positions that affect susceptibility of species to *Marburgvirus* and *Ebolavirus*, respectively [27]. Particular amino acids in positions 425 to 427 (TET) of loop-1 confer resistance to *Marburgvirus* in laboratory studies of cell lines; whereas residues at 502 (F) and 505 (T) of loop-2 confer resistance to *Ebolavirus* [27]. To test whether these regions of NPC1 loop 1 and 2 can predict the infection status of species that have been sampled for Ebolaviruses in the wild, we chose a window of 10 base pairs on either side of these amino acid positions identified by Takadate et al. [27] for statistical analyses of sequence variation. Of 307 mammal species for which NPC1 sequences were available, a total of 31 have been tested for *Ebolavirus* infection using antibodies or PCR.

<u>*Statistical analyses*</u>

Phylogenetic signal in response variables: We estimated phylogenetic signal in *Ebolavirus* infection status and mortality using the maximum clade credibility consensus tree from Upham et al. [21]. We measured phylogenetic signal using Fritz and Purvis' D for binary traits using the R package 'caper' and tested for significance of D assuming no phylogenetic structure and random Brownian process from phylogeny (e.g. [28]). Fritz and Purvis' D=1 or D>1 assumes binary traits that are random and overdispersed with respect to phylogeny. A value of 0 is expected for a Brownian model of evolution, and a value <0 for a phylogenetically conserved trait. Significance was tested by permuting binary traits across tips and recalculating D for each permutation, and by simulating traits along the phylogeny while assuming a Brownian process.

Statistical models: We used ridge regression with the R package 'ridge' [29,30] to model death of mammal hosts as a function of traits, distance of species range to nearest spillover site and the first 48 phylogenetic eigenvectors. Due to allometric scaling and evolutionary descent relationships, species traits tend to show high collinearity [31,32]. The ridge method is a penalized approach that typically performs well for multivariate analyses with correlated predictors [33]. However, for small sample sizes, selecting an appropriate shrinkage parameter (lambda) is challenging with methods that use cross-validation [29,34]. Our final dataset included only 21 species with any *Ebolavirus* mortality data, making cross-validation unreliable. We therefore used a modified procedure for selecting the ridge parameter that minimizes variance of predictions [29,34]. To assess model accuracy and quantify goodness-of-fit, we used leave-one-out cross validation to determine the fraction of observations correctly predicted by the model [35]. We predicted positive *Ebolavirus* infection status across different mammal species using trait variables, distance of species range to nearest spillover site, first 48 phylogenetic eigenvectors and sampling effort as predictors in a machine learning ridge regression framework. We tuned this ridge regression model parameters using the R packages 'caret' and 'glmnet' with repeated cross validation method, 5-fold with 5 repeats, downsampling to balance the design, and with area under the curve (AUC) as a performance measure. Across a range of lambda values 0-3, the parameter leading to the highest AUC value was selected as the shrinkage estimate. We then supplied the estimated lambda to the *logisticRidge*() command in R package 'ridge' to compute coefficient estimates, t-statistics, and accompanying *p*-values for all predictors [30].

To estimate the relative contribution of each variable to predicting infection status, we calculated the AUC of a null model with non-significant predictors and then sequentially added each additional predictor to examine the change in AUC and thus the relative contribution of each variable. We chose this method because of the high degree of correlation among predictor variables. Traditionally, variable importance is computed by permuting one variable and calculating the difference in the performance measure, such as AUC. However, in highly correlated datasets, permuting a single variable to calculate variable importance may not be informative [36–38], because other, correlated, variables can be significant and explain the outcome, producing no change in performance even after permutation. We started with a null model that included percent of diet from scavenging and percent diet from granivory as predictors, variables not significantly related to infection status in preliminary models. Null models of these two variables yielded an AUC=0.477 (i.e., no ability to discriminate 0 and 1). We then added each new predictor one at a time and calculated AUC on test datasets created using the 'caret' package using the repeated cross validation approach (5 folds, 5 repeats, lambda parameter determined by cross validation). To ensure that our final regression predictors were robust, we repeated the analysis for subsets of the data including antibody- and PCR-tested species with sample sizes of 10 or more, PCR-tested species only, and free-ranging individuals only (excluding captive specimens or domesticated taxa).

Using our final model, we predicted the reservoir status of all terrestrial African mammals. For the infection status model, we set sampling effort to be a constant for all species at ~100 individuals (the mean number of individuals sampled for each species across all studies). Once we predicted death and infection probabilities, we set the cutoff to the threshold value for recovering known mortality for the 21 species in the mortality dataset and known infection for the 363 species in the infection dataset. We used the R package 'OptimalCutpoints' to identify these thresholds [39] and applied them to define death and infection. We then created four categories of reservoir status: 1. low probability of death and low probability of infection, which we interpreted as 'low exposure and susceptibility'; 2. low probability of death and high probability of infection, which we interpreted as 'potential reservoirs'; 3. high probability of death and low probability of infection, which we interpreted as 'not exposed to *Ebolavirus*'; 4. high probability of death and high probability of infection, which we interpreted as 'dead-end hosts' succumbing to infection.

Finally, we tested whether amino acid residues from two loop regions of the NPC1 receptor protein were related to infection status. We did not fit a model of NPC1 amino acid residues to death after inoculation with *Ebolavirus* due to the small number of taxa for which both inoculation status and NPC1 sequences were available. We created dummy variables for each amino acid residue at a given position using the R package 'fastDummies'. We then predicted infection status by logistic ridge regression implemented in the R package 'ridge' [29,30]. To ensure that these results were robust, we repeated this regression with NPC1 sequences and nuisance variables of distance of species range to spillover site and sampling effort as predictors for infection status. To investigate agreement between NPC1 and trait-based models, we compared percent correct predictions for each mammal order by trait and phylogenetic eigenvector models and percent correct predictions made by our NPC1 amino acid residues model.

Robustness of results to phylogenetic uncertainty: We tested the sensitivity of our ridge regression models predicting mortality and infection status of mammalian hosts for uncertainty

in species phylogenetic relationships. We downloaded a sample of 100 random trees from the Bayesian posterior distribution of potential fully resolved trees provided by Upham et al. [21]. For each of these 100 trees, we computed phylogenetic eigenvalues and eigenvectors using R package 'PVR' [22]. We determined the number of eigenvectors needed to capture 75% of the phylogenetic relationships for each of the 100 trees. We then repeated all ridge regressions with R package 'ridge' using the same trait variables of (i) adult mass (g), (ii) brain mass (g), (iii) maximum longevity (d), (iv) age at first reproduction (d), (v) gestation length (d), (vi) litter size, (vii) litter per year, percent diet comprised of (viii) scavenging, (ix) granivory, (x) frugivory, (xi) plant material, (xii) distance of geographic range to a spillover site (m), and, in the case of antibody and PCR datasets estimating infection status of animal host, (xiii) sampling effort. We also included the number of phylogenetic eigenvectors required to explain 75% of the variance in phylogenetic relationships among species in each tree. Across all 100 trees and for each response variable of mortality or infection status, we summarized the proportion of the 100 runs where the predictor was significant and the predictive power of the model. We used leave-one-out cross validation method to obtain percent accuracy of our host mortality model and area under the curve or AUC for the infection status model. We also summarized across all 100 runs, the minimum, lower 2.5% quantile, mean, median, upper 97.5% quantile and maximum t-values for each predictor. The direction of eigenvectors changed across different trees from the posterior distribution of trees in Upham et al. [21] for the same mammal clades. Therefore, we summarized the distribution of absolute values of the t-statistic for eigenvectors. Finally, we estimated skewness of the histogram of t-values for each predictor, using raw t-statistics for traits and the absolute values of t-statistics for the phylogenetic eigenvectors.

Supplementary Results

Validity of ridge model predictors: Life history traits describing pace of life, percentage of fruit in diet and sampling effort consistently explained infection status as estimated by antibody and PCR tests. We tested sensitivity of ridge regression models to the inclusion of studies 1) in which species were poorly sampled, 2) reliant only on antibody tests and inclusion of captive, and 3) including domesticated species (Table 2 in main paper). Exclusion of studies with poor sampling or of domesticated or captive species lowered AUC values to approximately 0.76-0.77 (Table 1 in main paper). Across all regressions, infection status was found to be related to at least one pace of life trait including adult body mass, brain mass, maximum longevity, age at first reproduction, gestation length, litter size and number of litters per year (Table 2 in main paper). These life history traits had high relative importance in ridge regression models, improving AUC values from 0.17 to as much as 0.28 (S1 Table). Furthermore, 7 of the top 15 variables with the highest relative importances were traits related to pace of life (S2 Table). Percent fruit in diet was also a significant predictor of infection status across all sensitivity tests performed, even showing marginal significance at $\alpha=0.1$ in PCR tested individuals (Table 2 in main paper). Inclusion of percent fruit in diet as a predictor increased the AUC value by 0.2 (S2 Table). Finally, sampling effort had the highest relative importance of 0.34 (S2 Table) and was always a significant predictor of infection status (Table 2 in main paper).

Robustness of results to phylogenetic uncertainty: We found similar results across the random sample of 100 trees from the Bayesian posterior distribution of trees in Upham et al. [21]. The percent accuracy of our mortality model using leave-one-out cross validation method was on

average 0.86 (95% quantile interval is 0.71-0.94). The average AUC value of our infection status model across 100 trees was 0.81 (95% quantile interval is 0.80-0.82). Mortality of animal host was positively predicted by gestation length and negatively by litters per year (S3 Table). The third phylogenetic eigenvector (c3) which differentiates Primates and Artiodactyla from other clades like Chiroptera and Rodentia (S5A Fig) was also related to mortality (S3 Table). High infection probability occurred in species with high maximum longevity, high age at first reproduction, longer gestation lengths, few litters per year and small litter sizes (S4 Table). Percent fruit in diet was also positively related to infection status, as was sampling effort (S4 Table). Phylogenetic eigenvectors c3, c11 and c12 were also related to infection status (S4 Table). These eigenvectors separate order Chiroptera including the particular clade that carries Pteropodid bats (S5B Fig), Primates including the clade with families Cercopithecidae and Hominidae (S5C Fig), and broader groupings that differentiate Primates and Artiodactyla from other clades (S5A Fig).

Literature Cited in supplement

1. Bennett RS, Huzella LM, Jahrling PB, Bollinger L, Olinger Jr GG, Hensley LE. Nonhuman primate models of Ebola virus disease. In: Mühlberger E, Towner JS, Henley LL, editors. Marburg- and Ebolaviruses From Ecosysems to Molecules. Cham, Switerzland: Springer International; 2017. pp. 171–194.
2. Yamaoka S, Banadyga L, Bray M, Ebihara H. Small animal models for studying filovirus pathogenesis. In: Mühlberger E, Hensley LL, Towner JS, editors. Marburg- and Ebolaviruses From Ecosysems to Molecules. Cham, Switerzland: Springer International; 2017. pp. 195–227.
3. Bermejo M, Rodríguez-Teijeiro JD, Illera G, Barroso A, Vilà C, Walsh PD. Ebola outbreak killed 5000 gorillas. Science (80-). 2006;314: 1564. doi:10.1126/science.1133105
4. Formenty P, Boesch C, Wyers M, Steiner C, Donati F, Dind F, et al. Ebola virus outbreak among wild chimpanzees living in a rain forest of Cote d'Ivoire. J Infect Dis. 1999;179: 120–126. doi:10.1086/514296
5. Wittmann TJ, Biek R, Hassanin A, Rouquet P, Reed P, Yaba P, et al. Isolates of *Zaire ebolavirus* from wild apes reveal genetic lineage and recombinants. Proc Natl Acad Sci U S A. 2007;104: 19656. doi:10.1073/pnas.0710119104
6. Miranda ME, Ksiazek TG, Retuya TJ, Khan AS, Sanchez A, Fulhorst CF, et al. Epidemiology of Ebola (subtype Reston) virus in the Philippines, 1996. J Infect Dis. 1999;179: S115–S119. doi:10.1086/514314
7. Miranda MEG, Yoshikawa Y, Manalo DL, Calaor AB, Miranda NLJ, Fumiaki C, et al. Chronological and spatial analysis of the 1996 Ebola Reston Virus Outbreak in a Monkey Breeding Facility in the Phillipines. Exp Anim. 2002;51: 173–179.
8. World Health Organization. WHO experts consultation on Ebola Reston pathogenicity in humans. 2009; 1–22. Available: http://www.who.int/csr/resources/publications/WHO_HSE_EPR_2009_2/en/
9. Soria CD, Pacifici M, Di Marco M, Stephen SM, Rondinini C. COMBINE: a coalesced mammal database of intrinsic and extrinsic traits. Ecology. 2021;102: 13028255. doi:10.1002/ecy.3344
10. Faurby S, Davis M, Pedersen R, Schowanek SD, Antonelli A, Svenning JC. PHYLACINE 1.2: The phylogenetic atlas of mammal macroecology. Ecology. 2018;99: 2626.
11. Wilman H, Belmaker J, Simpson J, de la Rosa C, Rivadeneira MM, Jetz W. EltonTraits 1.0: Species-level foraging attributes of the world's birds and mammals. Ecology. 2014;95: 2027–2027. doi:10.1890/13-1917.1
12. Jones KE, Bielby J, Cardillo M, Fritz SA, O'Dell J, Orme CDL, et al. PanTHERIA: a species-level database of life history, ecology, and geography of extant and recently extinct mammals. Ecology. 2009;90: 2648–2648. doi:10.1890/08-1494.1
13. Santini L, Isaac NJB, Ficetola GF. TetraDENSITY: A database of population density estimates in terrestrial vertebrates. Glob Ecol Biogeogr. 2018;27: 787–791. doi:10.1111/geb.12756
14. Kissling WD, Dalby L, Fløjgaard C, Lenoir J, Sandel B, Sandom C, et al. Establishing macroecological trait datasets: Digitalization, extrapolation, and validation of diet preferences in terrestrial mammals worldwide. Ecol Evol. 2014;4: 2913–2930. doi:10.1002/ece3.1136

15. Promislow DE., Harvey PH. Living fast and dying young: a comparative analysis of life-history variation among mammals. J Zool. 1990;220: 417–437. doi:https://doi.org/10.1111/j.1469-7998.1990.tb04316.x
16. Schmidt JP, Maher S, Drake JM, Huang T, Farrell MJ, Han BA. Ecological indicators of mammal exposure to *Ebolavirus*. Philos Trans R Soc B Biol Sci. 2019;374. doi:10.1098/rstb.2018.0337
17. Han BA, Schmidt JP, Alexander LW, Bowden SE, Hayman DTS, Drake JM. Undiscovered Bat Hosts of Filoviruses. PLoS Negl Trop Dis. 2016;10: 1–10. doi:10.1371/journal.pntd.0004815
18. Han BA, Schmidt JP, Bowden SE, Drake JM. Rodent reservoirs of future zoonotic diseases. Proc Natl Acad Sci U S A. 2015;112: 7039–7044. doi:10.1073/pnas.1501598112
19. Barton RA, Capellini I. Maternal investment, life histories, and the costs of brain growth in mammals. Proc Natl Acad Sci U S A. 2011;108: 6169–6174. doi:10.1073/pnas.1019140108
20. Schmidt JP, Park AW, Kramer AM, Han BA, Alexander LW, Drake JM. Spatiotemporal fluctuations and triggers of ebola virus spillover. Emerg Infect Dis. 2017;23: 415–422. doi:10.3201/eid2303.160101
21. Upham NS, Esselstyn JA, Jetz W. Inferring the mammal tree: Species-level sets of phylogenies for questions in ecology, evolution, and conservation. PLoS Biology. 2019. doi:10.1371/journal.pbio.3000494
22. Santos T, Diniz-Filho JA, Bini TR e LM. Package “PVR.” 2012. pp. 1–13.
23. Pruitt KD, Tatusova T, Brown GR, Maglott DR. NCBI Reference Sequences (RefSeq): Current status, new features and genome annotation policy. Nucleic Acids Res. 2012;40: 130–135. doi:10.1093/nar/gkr1079
24. Coordinators NR. Database resources of the National Center for Biotechnology Information. Nucleic Acids Res. 2016;44: D7–D19. doi:10.1093/nar/gkv1290
25. Papadopoulos JS, Agarwala R. COBALT: Constraint-based alignment tool for multiple protein sequences. Bioinformatics. 2007;23: 1073–1079. doi:10.1093/bioinformatics/btm076
26. Larsson A. AliView: A fast and lightweight alignment viewer and editor for large datasets. Bioinformatics. 2014;30: 3276–3278. doi:10.1093/bioinformatics/btu531
27. Takadate Y, Kondoh T, Igarashi M, Maruyama J, Manzoor R, Ogawa H, et al. Niemann-Pick C1 Heterogeneity of Bat Cells Controls Filovirus Tropism. Cell Rep. 2020;30: 308-319.e5. doi:10.1016/j.celrep.2019.12.042
28. Fritz SA, Purvis A. Selectivity in mammalian extinction risk and threat types: A new measure of phylogenetic signal strength in binary traits. Conserv Biol. 2010;24: 1042–1051. doi:10.1111/j.1523-1739.2010.01455.x
29. Cule E, De Iorio M. Ridge regression in prediction problems: Automatic choice of the ridge parameter. Genet Epidemiol. 2013;37: 704–714. doi:10.1002/gepi.21750
30. Moritz S, Cule E, Frankowski D. ridge: Ridge regression with automatic selection of the penalty parameter. R package; 2022.
31. West GB, Brown JH. The origin of allometric scaling laws in biology from genomes to ecosystems: Towards a quantitative unifying theory of biological structure and organization. J Exp Biol. 2005;208: 1575–1592. doi:10.1242/jeb.01589
32. Felsenstein J. Phylogenies and the Comparative Method. Am Soc Nat. 1985;125: 1–15.
33. Frank LE, Friedman JH. A statistical view of some chemometrics regression tools.

Technometrics. 1993;35: 109–135. doi:10.1080/00401706.1993.10485033
34. Cule E, Vineis P, De Iorio M. Significance testing in ridge regression for genetic data. BMC Bioinformatics. 2011;12. doi:10.1186/1471-2105-12-372
35. Ugarte MD, Militino AF, Arnholt AT. Probability and statistics with R. 2nd ed. Boca Raton: CRC Press; 2016.
36. Auret L, Aldrich C. Empirical comparison of tree ensemble variable importance measures. Chemom Intell Lab Syst. 2011;105: 157–170. doi:10.1016/j.chemolab.2010.12.004
37. Strobl C, Boulesteix AL, Kneib T, Augustin T, Zeileis A. Conditional variable importance for random forests. BMC Bioinformatics. 2008;9: 1–11. doi:10.1186/1471-2105-9-307
38. Nicodemus KK, Malley JD. Predictor correlation impacts machine learning algorithms: Implications for genomic studies. Bioinformatics. 2009;25: 1884–1890. doi:10.1093/bioinformatics/btp331
39. López-Ratón M, Rodríguez-Álvarez MX, Suárez CC, Sampedro FG. R Package "OptimalCutpoints." J Stat Softw. 2014;61: 1–36. Available: http://www.jstatsoft.org/v61/i08/%0Ahttps://cran.r-project.org/package=OptimalCutpoints
