# Supplementary material for: Traits, phylogeny and host cell receptors predict Ebolavirus host status among African mammals
Source: PLoS Negl Trop Dis. 2022 Dec 21;16(12):e0010993. doi: 10.1371/journal.pntd.0010993 (PMC9815631; doi:10.1371/journal.pntd.0010993)
Supplement: S1 Table — (PDF) [file pntd.0010993.s002.pdf]

S1 Table for "Traits, phylogeny and host cell receptors predict *Ebolavirus* host status of African mammals"

Mekala Sundaram, John Paul Schmidt, Barbara A. Han, John M. Drake, Patrick R. Stephens

S1 Table. Relative importances of predictor variables in ridge models. Relative importance computed as change in AUC after addition of each variable to a null model of percent scavenging and percent seed in diet. The null model had AUC of 0.477 or no ability to discriminate between negative or positive infection states given the two predictors were not significantly associated with infection status.

Variable	Change in AUC
sample size	0.341
gestation length d	0.286
max longevity d	0.257
c28	0.252
age first reproduction d	0.240
c4	0.216
litter size n	0.215
c12	0.215
brain mass g	0.214
c13	0.211
percent fruit	0.200
litters per year n	0.199
c3	0.194
adult mass g	0.174
c7	0.168
c14	0.145
c11	0.143
c6	0.133
c23	0.098
c10	0.087
distance to spillover m	0.085
c38	0.080
c29	0.075
c15	0.074
c5	0.069
c17	0.069
c19	0.062
c43	0.058
c8	0.052

c18	0.038
c26	0.037
c37	0.036
c35	0.030
c1	0.029
c25	0.028
c45	0.026
c9	0.025
c34	0.024
c31	0.024
c46	0.023
c48	0.019
c20	0.017
c39	0.016
c44	0.012
c47	0.011
percent plant	0.010
c21	0.009
c30	0.001
c16	-0.001
terrestrial volant	-0.004
c22	-0.004
c41	-0.005
c2	-0.005
c24	-0.005
c36	-0.006
c42	-0.008
c33	-0.012
c32	-0.019
c40	-0.034
c27	-0.052
