# Supplementary material for: Traits, phylogeny and host cell receptors predict Ebolavirus host status among African mammals
Source: PLoS Negl Trop Dis. 2022 Dec 21;16(12):e0010993. doi: 10.1371/journal.pntd.0010993 (PMC9815631; doi:10.1371/journal.pntd.0010993)
Supplement: S2 Table — (PDF) [file pntd.0010993.s003.pdf]

S2 Table for "Traits, phylogeny and host cell receptors predict *Ebolavirus* host status of African mammals"

Mekala Sundaram, John Paul Schmidt, Barbara A. Han, John M. Drake, Patrick R. Stephens

S2 Table. Sensitivity of mortality of animal host model to uncertainty in phylogenetic relationships. We repeated our ridge regression model predicting mortality with trait variables and phylogenetic eigenvectors from 100 random trees from the Bayesian posterior distribution of trees in Upham et al (DOI: 10.1371/journal.pbio.3000494). Table summarizes for each predictor variable, the proportion of n=100 runs where the predictor was significantly related to mortality at α of 0.05. Table also summaries the t-statistics from all runs, providing the minimum, lower 2.5% quantile, mean, median, upper 97.5% quantile, maximum t-value and the skewness of the distribution. Note that the t-statistics for all the phylogenetic eigenvectors were summarized after calculating their absolute values, due to random assignment of directionality to mammal clades across different trees from the posterior distribution. A value of NA indicates lower phylogenetic eigenvectors that were used from only one posterior tree to account for 75% cumulative variation in phylogenetic information; therefore, a histogram of t-statistics cannot be computed from one value. Bold faced values represent predictors that were significantly related to mortality in 90% of repeats across trees.

Variables	proportion runs	min	l95	mean	median	u95	max	skew
adult_mass_g	0.000	-0.557	-0.529	0.009	-0.058	1.302	1.577	2.171
brain_mass_g	0.000	0.330	0.374	0.664	0.622	1.518	1.801	2.608
max_longevity_d	0.090	0.945	1.003	1.562	1.550	2.560	2.711	1.374
age_first_reproduction_d	0.090	1.051	1.099	1.493	1.457	2.437	2.617	2.013
**gestation_length_d**	**1.000**	**2.044**	**2.083**	**2.374**	**2.365**	**2.796**	**2.816**	**0.516**
litter_size_n	0.000	-0.816	-0.705	-0.267	-0.257	0.037	0.142	-0.522
**litters_per_year_n**	**0.950**	**-3.166**	**-3.122**	**-2.547**	**-2.567**	**-1.883**	**-1.809**	**0.331**
det_scav	0.000	0.615	0.740	0.982	0.985	1.249	1.365	0.024
det_seed	0.000	-0.720	-0.673	-0.513	-0.524	-0.322	-0.296	0.189
det_fruit	0.000	0.421	0.454	0.615	0.610	0.821	0.871	0.303
det_plantother	0.000	-1.596	-1.536	-1.305	-1.337	-0.749	-0.651	1.747
prox	0.000	-0.168	-0.115	0.291	0.262	1.224	1.474	2.011
**terrestrial_volant**	**1.000**	**-2.589**	**-2.504**	**-2.305**	**-2.310**	**-2.110**	**-2.083**	**-0.036**
c1	0.010	0.003	0.032	0.501	0.347	1.641	2.466	1.610
c2	0.190	0.008	0.064	1.055	0.883	2.469	2.692	0.545
**c3**	**1.000**	**2.016**	**2.211**	**3.029**	**3.083**	**3.643**	**3.748**	**-0.676**
c4	0.230	0.054	0.200	1.460	1.577	2.551	3.117	-0.178
c5	0.600	0.021	0.051	1.903	2.520	3.344	3.428	-0.507
c6	0.320	0.008	0.031	1.107	0.511	3.056	3.427	0.649
c7	0.040	0.003	0.007	0.311	0.191	2.268	2.852	3.700
c8	0.000	0.044	0.079	0.771	0.845	1.304	1.507	-0.333

c9	0.060	0.063	0.111	1.162	1.180	2.069	2.095	-0.230
c10	0.160	0.502	0.819	1.527	1.633	2.112	2.175	-0.348
c11	0.150	0.313	0.802	1.517	1.430	2.673	2.988	0.879
c12	0.780	0.404	1.261	2.366	2.521	3.223	3.313	-0.942
c13	0.220	0.055	0.123	1.278	1.427	2.768	3.069	0.194
c14	0.060	0.012	0.035	0.957	0.794	2.252	2.607	0.457
c15	0.020	0.006	0.058	0.883	0.736	1.731	2.340	0.208
c16	0.000	0.006	0.061	0.768	0.708	1.694	1.817	0.437
c17	0.000	0.001	0.034	0.785	0.812	1.469	1.607	-0.272
c18	0.000	0.050	0.103	0.766	0.795	1.254	1.445	-0.417
c19	0.000	0.005	0.074	0.844	0.874	1.547	1.891	-0.109
c20	0.010	0.031	0.071	0.928	0.925	1.748	2.127	0.000
c21	0.000	0.016	0.083	1.071	1.116	1.855	1.898	-0.466
c22	0.010	0.045	0.148	0.953	0.997	1.624	1.988	-0.282
c23	0.000	0.118	0.246	0.833	0.876	1.420	1.753	0.017
c24	0.010	0.249	0.442	0.971	0.895	1.658	1.993	0.437
c25	0.030	0.136	0.396	0.984	0.935	1.961	2.177	0.676
c26	0.020	0.146	0.197	0.941	0.881	1.731	2.079	0.502
c27	0.000	0.010	0.138	0.888	0.818	1.575	1.711	0.285
c28	0.000	0.028	0.112	0.807	0.749	1.579	1.836	0.354
c29	0.000	0.020	0.030	0.518	0.374	1.405	1.614	0.852
c30	0.020	0.032	0.364	1.193	1.239	1.859	2.188	-0.505
c31	0.020	0.034	0.203	1.132	1.066	1.906	2.353	-0.029
c32	0.000	0.004	0.224	1.141	1.141	1.763	1.864	-0.452
c33	0.000	0.053	0.095	0.873	0.856	1.821	1.907	0.196
c34	0.000	0.000	0.017	0.768	0.761	1.650	1.943	0.256
c35	0.000	0.022	0.039	0.636	0.543	1.501	1.601	0.494
c36	0.000	0.005	0.024	0.567	0.481	1.382	1.633	0.659
c37	0.010	0.017	0.041	0.688	0.660	1.617	2.006	0.532
c38	0.000	0.038	0.142	0.725	0.671	1.463	1.724	0.493
c39	0.000	0.043	0.085	0.833	0.839	1.552	1.749	-0.031
c40	0.000	0.011	0.094	0.846	0.856	1.708	1.802	0.154
c41	0.000	0.028	0.096	0.884	0.915	1.695	1.757	-0.079
c42	0.000	0.002	0.245	0.875	0.868	1.513	1.666	-0.024
c43	0.000	0.194	0.207	0.893	0.813	1.734	1.847	0.397
c44	0.000	0.028	0.100	0.929	0.878	1.714	1.859	0.032
c45	0.000	0.111	0.180	0.934	0.948	1.631	1.787	-0.022
c46	0.000	0.006	0.058	0.885	0.902	1.594	1.733	-0.146
c47	0.000	0.110	0.175	0.920	0.889	1.618	1.720	0.113
c48	0.000	0.070	0.142	0.859	0.898	1.568	1.623	-0.075

c49	0.000	0.405	0.454	0.907	0.875	1.497	1.606	0.484
c50	0.000	0.510	0.513	0.832	0.811	1.303	1.391	0.683
c51	0.000	0.418	0.471	0.963	1.038	1.264	1.278	-0.702
c52	0.000	0.950	0.957	1.103	1.084	1.281	1.294	0.258
c53	0.000	0.666	0.680	0.948	0.917	1.268	1.292	0.240
c54	0.000	NA	NA	NA	NA	NA	NA	NA
