# Supplementary material for: Traits, phylogeny and host cell receptors predict Ebolavirus host status among African mammals
Source: PLoS Negl Trop Dis. 2022 Dec 21;16(12):e0010993. doi: 10.1371/journal.pntd.0010993 (PMC9815631; doi:10.1371/journal.pntd.0010993)
Supplement: S3 Table — (PDF) [file pntd.0010993.s004.pdf]

S3 Table for "Traits, phylogeny and host cell receptors predict *Ebolavirus* host status of African mammals"

Mekala Sundaram, John Paul Schmidt, Barbara A. Han, John M. Drake, Patrick R. Stephens

S3 Table. Sensitivity of infection status model to uncertainty in phylogenetic relationships. We repeated our ridge regression model predicting mortality with trait variables and phylogenetic eigenvectors from 100 random trees from the Bayesian posterior distribution of trees in Upham et al (DOI: 10.1371/journal.pbio.3000494). Table summarizes for each predictor variable, the proportion of n=100 runs where the predictor was significantly related to mortality at α of 0.05. Table also summaries the t-statistics from all runs, providing the minimum, lower 2.5% quantile, mean, median, upper 97.5% quantile, maximum t-value and the skewness of the distribution. Note that the t-statistics for all the phylogenetic eigenvectors were summarized after calculating their absolute values, due to random assignment of directionality to mammal clades across different trees from the posterior distribution. A value of NA indicates lower phylogenetic eigenvectors that were used from only one posterior tree to account for 75% cumulative variation in phylogenetic information; therefore, a histogram of t-statistics cannot be computed from one value. Bold faced values represent predictors that were significantly related to mortality in at least 90% or more of the posterior trees.

Variable	proportion runs	min	l95	mean	median	u95	max	skew
adult_mass_g	0.890	1.615	1.809	2.522	2.565	3.226	3.239	-0.148
brain_mass_g	0.890	1.333	1.691	2.703	2.763	3.698	3.738	-0.258
**max_longevity_d**	**1.000**	**3.216**	**3.999**	**5.281**	**5.414**	**6.170**	**6.201**	**-1.046**
**age_first_reproduction_d**	**1.000**	**3.021**	**3.839**	**5.208**	**5.335**	**6.194**	**6.230**	**-0.926**
**gestation_length_d**	**1.000**	**3.737**	**4.561**	**5.797**	**5.973**	**6.492**	**6.532**	**-1.362**
**litter_size_n**	**1.000**	**-4.173**	**-4.157**	**-3.913**	**-3.945**	**-3.480**	**-3.167**	**1.247**
**litters_per_year_n**	**1.000**	**-4.207**	**-4.196**	**-3.619**	**-3.724**	**-2.646**	**-2.185**	**1.070**
det_scav	0.000	-1.510	-1.503	-1.352	-1.373	-1.134	-1.035	0.757
det_seed	0.000	-0.422	-0.416	-0.318	-0.332	-0.154	-0.108	0.720
**det_fruit**	**1.000**	**2.814**	**3.126**	**3.975**	**4.071**	**4.578**	**4.598**	**-0.711**
det_plantother	0.000	-0.387	-0.361	-0.135	-0.122	0.033	0.074	-0.422
prox	0.570	-2.094	-2.047	-1.969	-1.963	-1.916	-1.913	-0.884
terrestrial_volant	0.010	0.224	0.289	0.744	0.635	1.697	2.081	1.231
c1	0.560	0.481	0.674	2.068	2.043	3.769	4.098	0.298
c2	0.110	0.022	0.101	1.101	1.128	2.250	2.774	0.339
**c3**	**0.960**	**1.119**	**1.930**	**3.447**	**3.481**	**4.892**	**5.390**	**-0.158**
c4	0.850	0.101	0.747	3.679	3.858	6.219	6.343	-0.347
c5	0.690	0.001	0.097	3.755	4.791	6.247	6.333	-0.526
c6	0.470	0.040	0.068	2.433	1.629	6.077	6.339	0.422
c7	0.870	0.001	0.343	2.631	2.794	4.263	5.670	-0.824

c8	0.310	0.004	0.049	1.461	1.280	3.277	3.475	0.372
c9	0.460	0.071	0.191	1.926	1.809	3.678	3.937	0.100
c10	0.660	0.292	0.488	2.390	2.925	3.486	3.592	-0.647
**c11**	**1.000**	**2.392**	**2.693**	**3.884**	**3.608**	**6.376**	**6.619**	**1.293**
**c12**	**0.930**	**0.322**	**1.153**	**4.122**	**4.499**	**5.463**	**5.786**	**-1.153**
c13	0.720	0.066	0.374	2.603	2.304	4.953	5.227	0.249
c14	0.490	0.046	0.147	1.896	1.942	4.597	4.864	0.504
c15	0.490	0.026	0.100	1.593	1.748	3.907	4.785	0.422
c16	0.190	0.002	0.016	1.081	1.018	2.481	2.608	0.406
c17	0.080	0.012	0.035	0.964	0.881	2.415	2.744	0.506
c18	0.050	0.015	0.050	0.913	0.889	2.022	2.092	0.188
c19	0.020	0.000	0.029	0.657	0.529	1.834	2.449	0.970
c20	0.030	0.022	0.067	0.894	0.673	1.996	2.932	0.508
c21	0.010	0.003	0.018	0.646	0.459	1.654	2.049	0.888
c22	0.050	0.002	0.006	0.698	0.420	2.016	2.087	0.660
c23	0.020	0.013	0.032	0.943	0.969	1.818	6.288	3.124
c24	0.310	0.005	0.071	2.129	0.466	6.382	6.719	0.814
c25	0.220	0.003	0.025	1.577	0.394	6.419	6.777	1.368
c26	0.150	0.000	0.043	1.372	0.467	6.293	6.535	1.824
c27	0.180	0.004	0.025	1.642	0.870	6.493	6.762	1.501
c28	0.150	0.029	0.153	1.616	1.168	6.408	6.707	1.768
c29	0.000	0.074	0.215	1.077	1.150	1.673	1.895	-0.738
c30	0.020	0.058	0.163	0.684	0.303	1.630	4.183	2.169
c31	0.020	0.013	0.092	1.001	1.053	1.887	2.414	0.032
c32	0.010	0.009	0.122	0.851	0.700	1.845	2.181	0.433
c33	0.010	0.029	0.117	0.782	0.734	1.746	2.053	0.599
c34	0.020	0.014	0.081	0.825	0.775	1.876	2.310	0.617
c35	0.000	0.001	0.016	0.810	0.789	1.559	1.744	-0.050
c36	0.000	0.013	0.029	0.696	0.698	1.381	1.429	0.039
c37	0.000	0.003	0.016	0.642	0.616	1.683	1.787	0.612
c38	0.000	0.017	0.032	0.613	0.527	1.538	1.715	0.578
c39	0.000	0.004	0.017	0.586	0.493	1.510	1.547	0.514
c40	0.000	0.012	0.058	0.670	0.566	1.534	1.768	0.516
c41	0.020	0.045	0.093	0.806	0.766	1.828	1.986	0.494
c42	0.010	0.008	0.037	0.682	0.623	1.672	2.089	0.647
c43	0.000	0.018	0.047	0.670	0.583	1.526	1.737	0.488
c44	0.020	0.020	0.029	0.624	0.539	1.869	2.120	1.015
c45	0.010	0.016	0.021	0.618	0.518	1.737	2.225	0.881
c46	0.000	0.001	0.003	0.519	0.375	1.763	1.832	1.024
c47	0.000	0.021	0.058	0.597	0.544	1.723	1.839	0.724

c48	0.000	0.012	0.017	0.534	0.371	1.696	1.842	1.134
c49	0.000	0.073	0.077	0.547	0.564	1.225	1.275	0.414
c50	0.000	0.049	0.082	0.675	0.669	1.419	1.496	0.344
c51	0.000	0.076	0.106	0.642	0.654	1.195	1.231	0.037
c52	0.000	0.595	0.607	0.830	0.812	1.084	1.101	0.171
c53	0.000	0.314	0.340	0.601	0.683	0.723	0.724	-0.706
c54	0.000	NA	NA	NA	NA	NA	NA	NA
**Freq**	**1.000**	**4.347**	**4.565**	**5.462**	**5.500**	**6.492**	**6.535**	**0.052**
