# Supplementary material for: Traits, phylogeny and host cell receptors predict Ebolavirus host status among African mammals
Source: PLoS Negl Trop Dis. 2022 Dec 21;16(12):e0010993. doi: 10.1371/journal.pntd.0010993 (PMC9815631; doi:10.1371/journal.pntd.0010993)
Supplement: S4 Table — (PDF) [file pntd.0010993.s005.pdf]

S4 Table for "Traits, phylogeny and host cell receptors predict *Ebolavirus* host status of African mammals"

Mekala Sundaram, John Paul Schmidt, Barbara A. Han, John M. Drake, Patrick R. Stephens

S4 Table. Ridge regression predicting infection status of animal host on the basis of Niemann-Pick C1 animal acid residues, distance to spillover site (m) and sampling effort. Results provided for two models: one based on NPC1, distance to spillover site and sampling effort; the other based only on NPC1 residues. For each predictor, a coefficient estimate is provided, along with accompanying t-value and p-value. Bold faced predictors are significant at α of 0.05. Italicized predictors are significant at α of 0.1.

	**NPC1 with distance to spillover and sampling effort**			**NPC1 sequences**		
	Estimate	t value	Pr(>|t|)	Estimate	t value	Pr(>|t|)
P410_Q	-0.116	-0.212	0.832	-0.246	-0.390	0.697
P410_R	0.116	0.212	0.832	0.246	0.390	0.697
P413_P	-0.418	-1.467	0.143	-0.429	-1.354	0.176
P413_T	0.418	1.467	0.143	0.429	1.354	0.176
P414_H	-0.279	-0.969	0.333	-0.339	-1.086	0.277
P414_L	0.104	0.514	0.607	0.132	0.618	0.537
P414_N	-0.045	-0.157	0.875	-0.032	-0.098	0.922
P414_Y	0.577	1.372	0.170	0.647	1.411	0.158
P415_N	0.418	1.467	0.143	0.429	1.354	0.176
P415_S	-0.468	-0.908	0.364	-0.560	-0.984	0.325
P415_T	0.139	0.311	0.756	0.209	0.424	0.671
P416_A	0.268	0.655	0.513	0.281	0.622	0.534
P416_D	0.178	0.517	0.605	0.189	0.495	0.621
P416_K	0.577	1.372	0.170	0.647	1.411	0.158
**P416_P**	**-0.864**	**-2.035**	**0.042**	**-0.945**	**-2.010**	**0.044**
P416_Q	0.418	1.467	0.143	0.429	1.354	0.176
P416_S	-0.355	-1.079	0.281	-0.346	-0.934	0.350
P417_I	0.435	0.767	0.443	0.376	0.587	0.557
P417_K	0.104	0.514	0.607	0.132	0.618	0.537
P417_M	0.631	1.215	0.225	0.743	1.296	0.195
P417_P	-0.667	-1.426	0.154	-0.732	-1.406	0.160
P417_S	0.418	1.467	0.143	0.429	1.354	0.176
P417_T	-0.262	-0.730	0.465	-0.338	-0.869	0.385
P417_V	-0.237	-0.644	0.520	-0.207	-0.507	0.612
P418_H	-0.577	-1.372	0.170	-0.647	-1.411	0.158
P418_S	0.577	1.372	0.170	0.647	1.411	0.158
P419_I	-0.194	-0.289	0.773	-0.185	-0.242	0.809

P419_K	0.652	1.047	0.295	0.735	1.066	0.286
P419_T	-0.078	-0.118	0.906	-0.120	-0.160	0.872
P421_E	-0.240	-0.674	0.500	-0.172	-0.435	0.663
P421_H	-0.036	-0.095	0.924	-0.090	-0.215	0.830
P421_Q	0.243	0.724	0.469	0.208	0.561	0.575
**P425_A**	**1.107**	**2.617**	**0.009**	**1.253**	**2.660**	**0.008**
P425_S	-0.566	-1.522	0.128	-0.576	-1.414	0.157
**P425_T**	*-0.611*	*-1.722*	*0.085*	**-0.790**	**-2.061**	**0.039**
**P426_E**	*-0.611*	*-1.722*	*0.085*	**-0.790**	**-2.061**	**0.039**
**P426_G**	*0.611*	*1.722*	*0.085*	**0.790**	**2.061**	**0.039**
P427_A	-0.165	-0.711	0.477	-0.117	-0.469	0.639
P427_S	*0.549*	*1.797*	*0.072*	*0.610*	*1.839*	*0.066*
**P427_T**	*-0.611*	*-1.722*	*0.085*	**-0.790**	**-2.061**	**0.039**
P433_A	-0.468	-0.960	0.337	-0.553	-1.032	0.302
P433_P	0.468	0.960	0.337	0.553	1.032	0.302
P436_A	-0.487	-1.078	0.281	-0.462	-0.909	0.363
P436_D	0.083	0.240	0.810	0.083	0.221	0.825
P436_N	0.014	0.036	0.971	-0.005	-0.012	0.991
P436_S	0.418	1.467	0.143	0.429	1.354	0.176
P437_I	*0.581*	*1.767*	*0.077*	*0.621*	*1.703*	*0.089*
P437_K	-0.639	-1.512	0.131	-0.701	-1.474	0.140
P437_R	0.012	0.025	0.980	0.028	0.052	0.959
P437_V	-0.179	-0.392	0.695	-0.179	-0.349	0.727
P490_D	-0.057	-0.114	0.909	-0.025	-0.044	0.965
P490_E	0.391	1.384	0.166	0.389	1.268	0.205
P490_G	-0.469	-1.241	0.215	-0.492	-1.168	0.243
P490_N	-0.179	-0.392	0.695	-0.179	-0.349	0.727
P490_Q	-0.065	-0.277	0.782	-0.067	-0.267	0.789
P491_N	0.382	0.915	0.360	0.485	1.063	0.288
P491_S	-0.382	-0.915	0.360	-0.485	-1.063	0.288
P493_A	-0.355	-1.079	0.281	-0.346	-0.934	0.350
P493_L	0.382	0.915	0.360	0.485	1.063	0.288
P493_S	0.067	0.188	0.851	-0.002	-0.006	0.995
P494_L	-0.523	-0.887	0.375	-0.567	-0.861	0.389
P494_M	0.642	1.606	0.108	0.693	1.578	0.114
P494_T	-0.338	-0.981	0.327	-0.441	-1.198	0.231
P494_V	-0.129	-0.483	0.629	-0.086	-0.295	0.768
P496_D	*-0.781*	*-1.744*	*0.081*	-0.799	-1.602	0.109
P496_N	*0.781*	*1.744*	*0.081*	0.799	1.602	0.109
P497_H	0.355	1.079	0.281	0.346	0.934	0.350

P497_N	-0.211	-0.395	0.693	-0.196	-0.328	0.743
P497_S	-0.283	-0.536	0.592	-0.286	-0.484	0.628
P498_E	*-0.669*	*-1.772*	*0.076*	*-0.740*	*-1.779*	*0.075*
P498_K	0.560	1.515	0.130	0.606	1.473	0.141
P498_Q	-0.362	-1.204	0.229	-0.334	-0.989	0.323
P498_T	0.435	0.767	0.443	0.376	0.587	0.557
P499_I	0.165	0.711	0.477	0.117	0.469	0.639
P499_K	0.104	0.514	0.607	0.132	0.618	0.537
P499_V	-0.362	-1.204	0.229	-0.334	-0.989	0.323
P502_A	-0.523	-0.887	0.375	-0.567	-0.861	0.389
P502_D	0.086	0.200	0.841	0.124	0.266	0.790
P502_F	*0.575*	*1.691*	*0.091*	*0.657*	*1.766*	*0.077*
P502_P	-0.439	-0.730	0.466	-0.583	-0.869	0.385
P504_F	0.380	0.931	0.352	0.525	1.150	0.250
P504_Y	-0.380	-0.931	0.352	-0.525	-1.150	0.250
P505_I	-0.384	-0.690	0.490	-0.418	-0.663	0.507
P505_T	-0.014	-0.028	0.978	0.069	0.120	0.905
P505_V	0.291	0.766	0.444	0.256	0.607	0.544
P513_F	0.469	1.241	0.215	0.492	1.168	0.243
P513_L	-0.469	-1.241	0.215	-0.492	-1.168	0.243
P516_C	0.179	0.392	0.695	0.179	0.349	0.727
P516_W	-0.179	-0.392	0.695	-0.179	-0.349	0.727
**Distance**	**-2.341**	**-2.599**	**0.009**			
Sample size	0.008	0.009	0.993			
