# Supplementary material for: Traits, phylogeny and host cell receptors predict Ebolavirus host status among African mammals
Source: PLoS Negl Trop Dis. 2022 Dec 21;16(12):e0010993. doi: 10.1371/journal.pntd.0010993 (PMC9815631; doi:10.1371/journal.pntd.0010993)
Supplement: S5 Table — (PDF) [file pntd.0010993.s006.pdf]

S5 Table for "Traits, phylogeny and host cell receptors predict *Ebolavirus* host status of African mammals"

Mekala Sundaram, John Paul Schmidt, Barbara A. Han, John M. Drake, Patrick R. Stephens

S5 Table. NPC1 residues at key positions identified by Takadate et al. (DOI: 10.1016/j.celrep.2019.12.042) for species for whom infection status is also known. Amino acid residues at positions 425-427 (P425-P427) are believed to confer resistance to *Marburgvirus* and residues in 502 and 505 (P502 and P505) are believed to confer resistance to *Ebolavirus*. Column 'infection status' represents known infection status determined from studies with antibody and PCR tests. Column 'NPC1 model predictions' provides estimated 0 and 1 predictions for species infection status based on a ridge regression model with species NPC1 sequences as predictors.

Species	P425	P426	P427	P502	P505	Infection status	NPC1 model predictions
Eidolon_helvum	A	G	S	F	T	1	1
Rousettus_aegyptiacus	A	G	S	D	V	1	1
Rousettus_leschenaultii	A	G	S	D	V	1	1
Miniopterus_schreibersii	S	G	S	D	V	1	1
Rhinolophus_ferrumequinum	S	G	S	D	V	1	1
Mus_musculus	A	G	A	D	I	0	0
Homo_sapiens	S	G	A	D	V	1	1
Rattus_norvegicus	S	G	A	D	V	0	0
Bos_taurus	S	G	A	D	V	0	0
Macaca_mulatta	S	G	A	D	V	0	1
Pan_troglodytes	S	G	A	D	V	1	1
Sus_scrofa	A	G	A	F	V	1	1
Pteropus_alecto	T	E	T	D	V	0	0
Artibeus_jamaicensis	S	G	S	P	V	0	0
Desmodus_rotundus	S	G	S	A	V	0	0
Sapajus_apella	S	G	A	D	V	0	0
Cercocebus_atys	S	G	A	D	V	1	1
Macaca_fascicularis	S	G	A	D	V	0	1
Macaca_nemestrina	S	G	A	D	V	0	1
Papio_anubis	S	G	A	D	V	1	1
Mandrillus_leucophaeus	S	G	A	D	V	1	1
Gorilla_gorilla	S	G	A	D	V	1	1
Pan_paniscus	S	G	A	D	V	0	1
Ovis_aries	S	G	A	D	V	0	0
Molossus_molossus	S	G	S	D	V	0	0

Myotis_myotis	S	G	S	D	I	0	0
Pipistrellus_kuhlii	S	G	S	D	T	0	0
Chlorocebus_sabaeus	S	G	A	D	V	0	1
Pteropus_vampyrus	T	E	T	D	V	0	0
Pteropus_giganteus	T	E	T	D	V	0	0
Hipposideros_armiger	A	G	S	D	V	1	1
