# Supplementary material for: Traits, phylogeny and host cell receptors predict Ebolavirus host status among African mammals
Source: PLoS Negl Trop Dis. 2022 Dec 21;16(12):e0010993. doi: 10.1371/journal.pntd.0010993 (PMC9815631; doi:10.1371/journal.pntd.0010993)
Supplement: S6 Table — (PDF) [file pntd.0010993.s007.pdf]

**S6 Table for "Traits, phylogeny and host cell receptors predict *Ebolavirus* host status of African mammals"**

Mekala Sundaram, John Paul Schmidt, Barbara A. Han, John M. Drake, Patrick R. Stephens

**S6 Table.** True positive rates (or sensitivity) and true negative rates (or specificity) provided for trait and phylogenetic eigenvector ridge model predicting infection status by mammalian clade. For each mammal group, the proportion of correct predictions, sensitivity, specificity and number of species tested are provided. If true positive rate is NA then all species tested within the clade were negative for *Ebolavirus* infection.

| Mammal group | Proportion correct predictions | True positive rate | True negative rate | Number of species tested |
|---|---|---|---|---|
| *Families:* | | | | |
| Pteropodidae | 0.485 | 1.000 | 0.056 | 33 |
| Cricetidae | 1.000 | NA | 1.000 | 1 |
| Cercopithecidae | 0.537 | 1.000 | 0.000 | 41 |
| Atelidae | 0.000 | NA | 0.000 | 1 |
| Anomaluridae | 1.000 | NA | 1.000 | 1 |
| Muridae | 1.000 | NA | 1.000 | 52 |
| Phyllostomidae | 1.000 | NA | 1.000 | 19 |
| Hipposideridae | 0.524 | 0.250 | 0.588 | 21 |
| Hystricidae | 1.000 | NA | 1.000 | 2 |
| Vespertilionidae | 0.841 | 0.000 | 0.902 | 44 |
| Sciuridae | 1.000 | NA | 1.000 | 15 |
| Canidae | 1.000 | NA | 1.000 | 3 |
| Megadermatidae | 1.000 | NA | 1.000 | 3 |
| Bovidae | 1.000 | NA | 1.000 | 6 |
| Molossidae | 0.889 | 0.000 | 1.000 | 18 |
| Emballonuridae | 1.000 | NA | 1.000 | 7 |
| Nesomyidae | 1.000 | NA | 1.000 | 5 |
| Soricidae | 0.952 | 0.000 | 1.000 | 21 |
| Herpestidae | 1.000 | NA | 1.000 | 2 |
| Procaviidae | 1.000 | NA | 1.000 | 1 |
| Erinaceidae | 1.000 | NA | 1.000 | 1 |
| Viverridae | 1.000 | NA | 1.000 | 3 |
| Hominidae | 0.600 | 1.000 | 0.000 | 5 |
| Gliridae | 1.000 | NA | 1.000 | 1 |
| Hylobatidae | 0.000 | NA | 0.000 | 1 |
| Miniopteridae | 0.667 | 0.000 | 1.000 | 6 |
| Mustelidae | 1.000 | NA | 1.000 | 1 |

| | | | | |
|---|---|---|---|---|
| Nandiniidae | 1.000 | NA | 1.000 | 1 |
| Nycteridae | 1.000 | NA | 1.000 | 4 |
| Lorisidae | 1.000 | NA | 1.000 | 2 |
| Ochotonidae | 1.000 | NA | 1.000 | 1 |
| Macroscelididae | 1.000 | NA | 1.000 | 2 |
| Suidae | 0.500 | 1.000 | 0.000 | 2 |
| Mormoopidae | 1.000 | NA | 1.000 | 3 |
| Rhinolophidae | 0.917 | 0.333 | 1.000 | 24 |
| Rhinopomatidae | 1.000 | NA | 1.000 | 2 |
| Spalacidae | 1.000 | NA | 1.000 | 2 |
| Callitrichidae | 0.000 | NA | 0.000 | 1 |
| Cebidae | 0.000 | NA | 0.000 | 1 |
| Thryonomyidae | 1.000 | NA | 1.000 | 1 |
| Tupaiidae | 1.000 | NA | 1.000 | 2 |
| Ursidae | 0.000 | NA | 0.000 | 1 |
| *Orders:* | | | | |
| Chiroptera | 0.783 | 0.586 | 0.819 | 184 |
| Rodentia | 1.000 | NA | 1.000 | 80 |
| Primates | 0.519 | 1.000 | 0.074 | 52 |
| Carnivora | 0.909 | NA | 0.909 | 11 |
| Cetartiodactyla | 0.857 | 1.000 | 0.833 | 7 |
| Eulipotyphla | 0.955 | 0.000 | 1.000 | 22 |
| Hyracoidea | 1.000 | NA | 1.000 | 1 |
| Lagomorpha | 1.000 | NA | 1.000 | 1 |
| Artiodactyla | 1.000 | NA | 1.000 | 1 |
| Macroscelidea | 1.000 | NA | 1.000 | 2 |
| Scandentia | 1.000 | NA | 1.000 | 2 |
