# Supplementary material for: Traits, phylogeny and host cell receptors predict Ebolavirus host status among African mammals
Source: PLoS Negl Trop Dis. 2022 Dec 21;16(12):e0010993. doi: 10.1371/journal.pntd.0010993 (PMC9815631; doi:10.1371/journal.pntd.0010993)
Supplement: S1 Fig — Phylogenetic eigenvector 3 or c3 plotted on maximum clade credibility tree (A), phylogenetic eigenvector 11 or c11 plotted on maximum clade credibility tree (B), and phylogenetic eigenvector 12 or c12 plotted on maximum clade credibility tree (C). These eigen scores consistently predict host mortality and host infection status; with c3 positively being related to host status, c11 and c12 being negatively related to host status. (PDF) [file pntd.0010993.s008.pdf]

S1 Fig for "Traits, phylogeny and host cell receptors predict *Ebolavirus* host status of African mammals"

Mekala Sundaram, John Paul Schmidt, Barbara A. Han, John M. Drake, Patrick R. Stephens

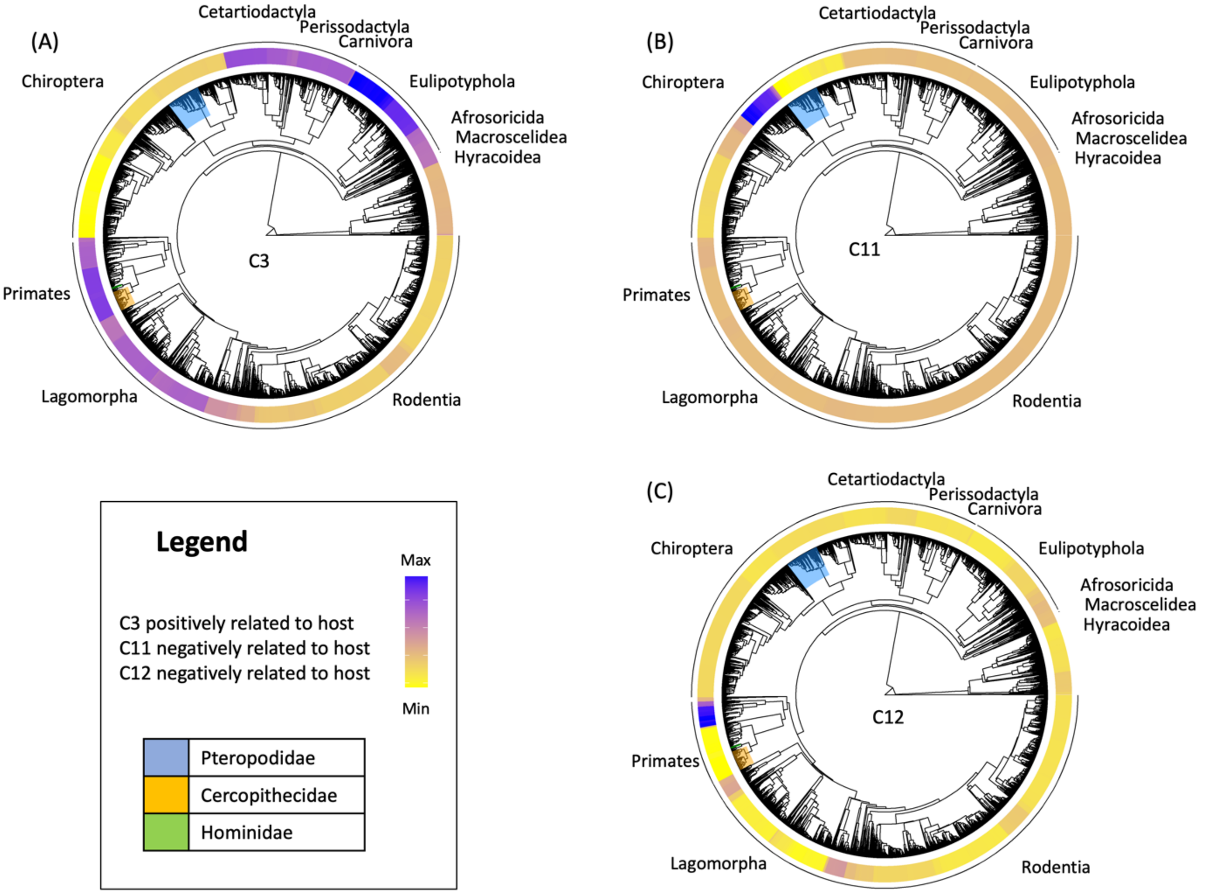

S1 Fig. Plots of significant phylogenetic eigenscores for all mammalian clades. Phylogenetic eigenvector 3 or c3 plotted on maximum clade credibility tree (A), phylogenetic eigenvector 11 or c11 plotted on maximum clade credibility tree (B), and phylogenetic eigenvector 12 or c12 plotted on maximum clade credibility tree (C). These eigen scores consistently predict host mortality and host infection status; with c3 positively being related to host status, c11 and c12 being negatively related to host status.
