# Supplementary material for: Traits, phylogeny and host cell receptors predict Ebolavirus host status among African mammals
Source: PLoS Negl Trop Dis. 2022 Dec 21;16(12):e0010993. doi: 10.1371/journal.pntd.0010993 (PMC9815631; doi:10.1371/journal.pntd.0010993)
Supplement: S2 Fig — (A) Predictions of reservoir status which are based on ridge model predicting mortality of species after exposure to Ebolavirus and ridge model predicting infection status of species. Ridge models used trait and phylogenetic eigenvectors as predictors (see main text for more details of models). (B) Accuracy of infection status predictions by mammal clade. Silhouettes used are available under Public Domain (https://creativecommons.org/publicdomain/zero/1.0/). (PDF) [file pntd.0010993.s009.pdf]

S2 Fig for “Traits, phylogeny and host cell receptors predict *Ebolavirus* host status of African mammals”

Mekala Sundaram, John Paul Schmidt, Barbara A. Han, John M. Drake, Patrick R. Stephens

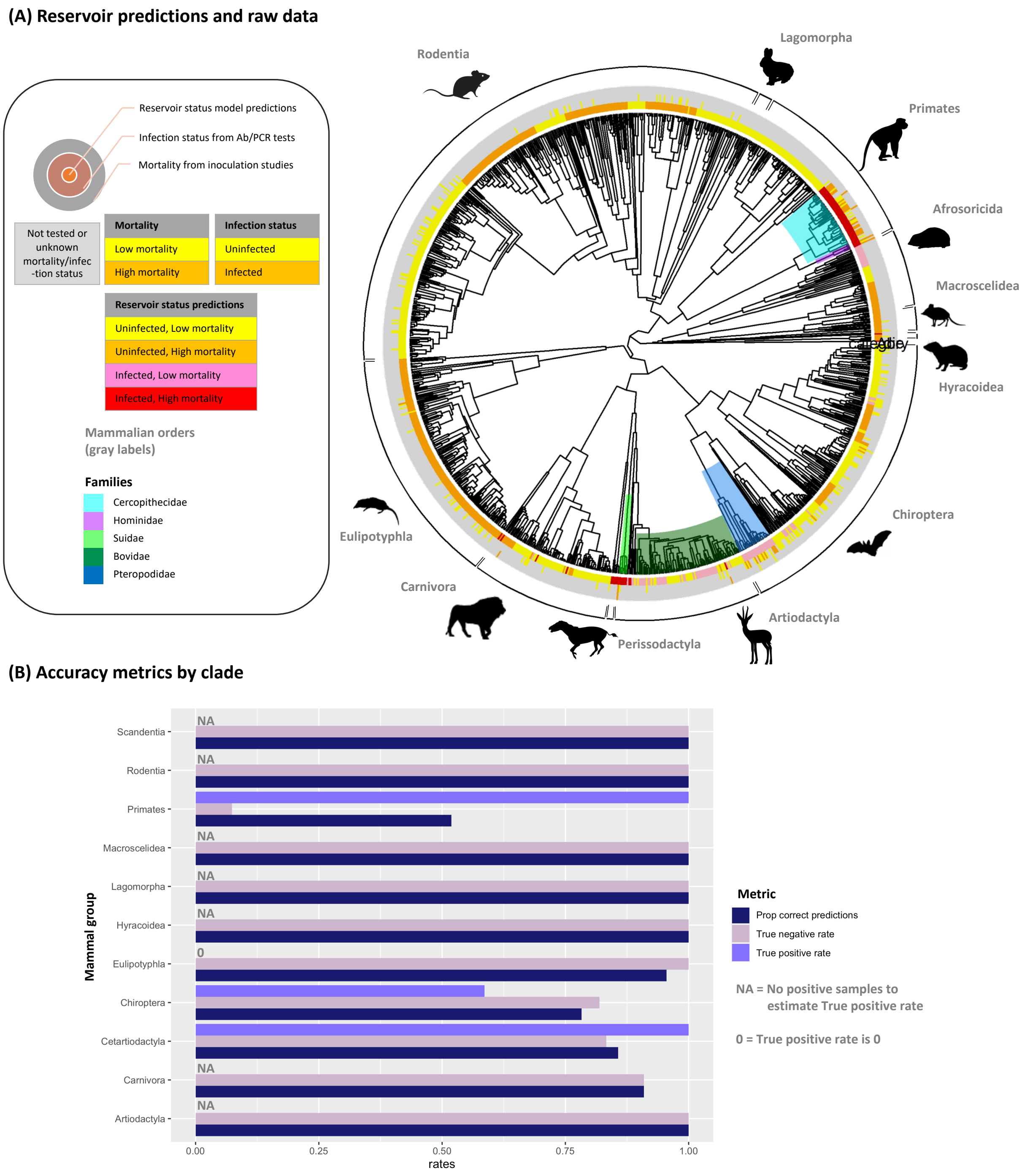

S2 Fig. Predictions of reservoir status for all known terrestrial African mammals, raw data showing infection status and mortality results for tested species (A). Predictions of reservoir status are based on ridge model predicting mortality of species after exposure to *Ebolavirus* and

ridge model predicting infection status of species. Ridge models used trait and phylogenetic eigenvectors as predictors (see main text for more details of models). Accuracy of infection status predictions by mammal clade (B). Silhouettes used are available under Public Domain (https://creativecommons.org/publicdomain/zero/1.0/).
